# Enhanced heparosan biosynthesis in *Corynebacterium glutamicum* by systematic metabolic engineering and translation-level fine-tuning

**DOI:** 10.1186/s40643-026-01113-5

**Published:** 2026-08-17

**Authors:** Jie Zhang, Yanan Cui, Peiyi Zhang, Mengqi Zhao, Liqiang Fan, Xu Li, Chen Deng, Liming Zhao

**Affiliations:** 1 State Key Laboratory of Bioreactor Engineering, School of Biotechnology, East China University of Science and Technology, Shanghai, 200237 China; 2 Shanghai Collaborative Innovation Center for Biomanufacturing Technology (SCICBT), Shanghai, 200237 China

**Keywords:** Heparosan, *Corynebacterium glutamicum*, Systematic metabolic engineering, Translation-level fine-tuning, Fed-batch fermentation

## Abstract

As a widely used anticoagulant, heparin is industrially produced chiefly via animal tissue extraction, which suffers from unstable supply and potential safety hazards. Heparosan shares a similar polysaccharide backbone with heparin and can be converted into heparin under mild enzymatic catalysis. In addition, heparosan exhibits favorable biocompatibility and non-immunogenicity, rendering its efficient, eco-friendly biosynthesis essential. In this study, we systematically engineered *Corynebacterium glutamicum*, a Generally Recognized as Safe (GRAS) microorganism, to synthesize heparosan via two complementary strategies. First, genome-scale modification was implemented to stably upregulate genes *ugd*, *glmS*, and *ndk*. The heparosan titer of recombinant strain Cg24 increased from 226.37 to 595.39 mg/L. Second, translation-level fine-tuning was implemented to modulate expression of individual genes within the *kfiB-kfiC-kfiA* cassette by constructing a high-coverage random ribosome binding site (RBS) library, which further lifted heparosan titer to 1161.37 mg/L. In fed-batch fermentation using a 5 L bioreactor with a two-stage growth-production regulation strategy, the recombinant strain Cg24-11 produced 5.36 g/L of heparosan, demonstrating its great potential for efficient heparosan biosynthesis. This combined modification strategy also provides valuable references for constructing high-efficiency cell factories for other complex compounds.

**Graphical abstract:**

**Figure:**
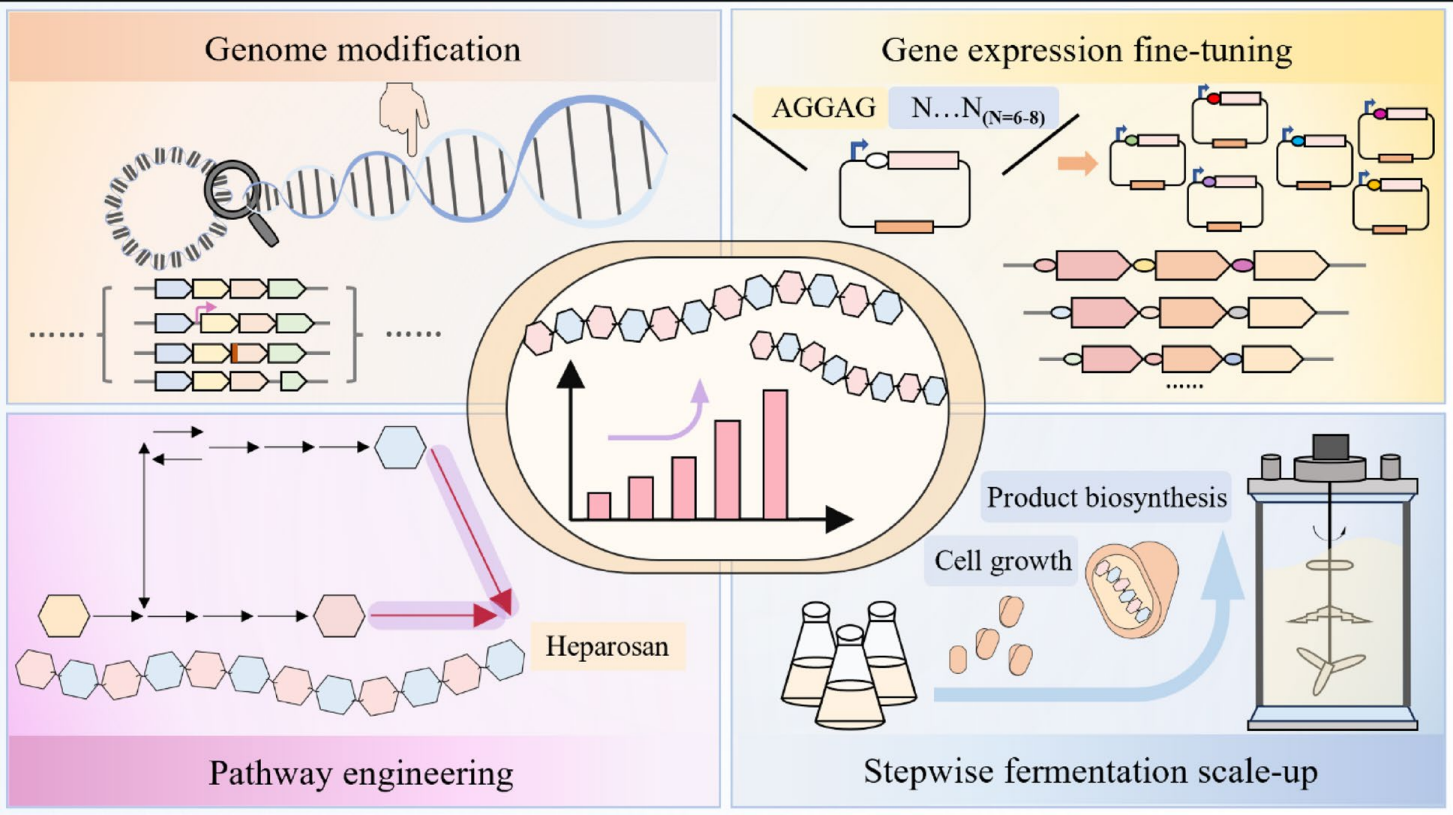

**Supplementary Information:**

The online version contains supplementary material available at 10.1186/s40643-026-01113-5.

## Introduction

As an important member of the glycosaminoglycan family, heparin possesses multiple desirable physiological functions including anticoagulation, antiviral activity and antitumor effects (Hogwood et al. 2023), and therefore it has extensive applications in medicine, clinical therapy and bio-based materials (Shi et al. 2021; Wang et al. 2022; Wu et al. 2025). However, industrial heparin is still predominantly extracted from animal tissues. Though technically mature, this approach may be restricted by livestock supply and entails potential safety and quality risks (Normile 2018). If safety hazards occur in heparin medicines, both consumers and production enterprises will suffer severe and far-reaching adverse impacts (Guerrini et al. 2008). Given this concern, an increasing number of alternative synthesis strategies have been explored to manufacture heparin and its analogues (Pan et al. 2026; Zhang et al. 2022). Heparosan, which shares a polysaccharide backbone analogous to heparin, can be transformed into bioengineered heparin via a cascade of enzymatic reactions (Baytas and Linhardt 2020; Jiang et al. 2025), offering a promising green alternative for sustainable heparin production (Sheng et al. 2024). Recent studies have revealed that, beyond serving as a substrate for heparin synthesis, heparosan also possesses various physiological activities such as maintaining intracellular environment stability and participating in the regulation of cell proliferation and differentiation. Its excellent biocompatibility and non-immunogenicity make heparosan an attractive vector for targeted drug delivery (Chen et al. 2015; Qiu et al. 2020; Rippe et al. 2019; Yang et al. 2022). Given these outstanding advantages, the efficient synthesis of heparosan has become a pressing necessity.

Nevertheless, the majority of native heparosan producers are pathogenic, represented by *Escherichia coli* K5 (Liu et al. 2012; Wang et al. 2010, 2011) and *Pasteurella multocida* type D (DeAngelis and White 2002). Existing engineering strategies remain relatively limited and lack diversity. Meanwhile, the products derived from these strains carry potential safety hazards, which greatly restricts heparosan clinical translation. Owing to the availability of microbial omics data and the development of gene editing and metabolic regulation technologies (Baytas and Linhardt 2020; Wan et al. 2023), heparosan can now be produced by safe microorganisms including *Bacillus subtilis* (Chen et al. 2017; Jin et al. 2016), *Bacillus megaterium* (Nehru et al. 2020) and *Corynebacterium glutamicum* (Yu et al. 2025) through heterologous expression of glycosyltransferase genes such as *kfiA*, *kfiC* or *PmHS*. Among these, *C. glutamicum*, a major industrial chassis for amino acid production (Zhao et al. 2025), supports high-density cultivation, features a diverse repertoire of gene editing and regulatory tools (Deng et al. 2024), and has mature industrial applications. Moreover, existing studies have shown that *C. glutamicum* enables efficient biosynthesis and chain elongation of glycosaminoglycans through its “membrane shield” strategy (Hu et al. 2022a; Shi et al. 2026). For these merits, *C. glutamicum* represents a highly promising chassis for the biosynthesis of heparosan.

For microbial heparosan synthesis, the polysaccharide chain is elongated alternately with UDP-GlcA and UDP-GlcNAc. Enzymes KfiA, KfiC and complex-stabilizing protein KfiB dominate the polymerization process, whereas the biosynthesis of the two UDP-sugar precursors relies on the coordinated expression of multiple genes to achieve sequential catalysis. Therefore, the expression levels of key biosynthetic genes determine both the titer and molecular weight of heparosan, and plasmid-mediated overexpression of these genes has become the prevailing strategy to boost intracellular UDP-sugar pools in recent research. By employing a two-plasmid system to overexpress the crucial genes involved in UDP-precursor biosynthesis, the heparosan titer in *Escherichia coli* Nissle 1917 reached 11.5 g/L in a 3 L bioreactor (Hu et al. 2022b). The same strategy was applied to *Bacillus megaterium*, resulting in a heparosan titer of 627 mg/L in 2 L fed-batch fermentation (Nehru et al. 2021). However, this approach also brings obvious drawbacks, including the risk of plasmid loss and heavy reliance on multiple antibiotics for strain screening (Chen et al. 2025). In comparison, genome-scale modification offers higher genetic stability and lower production costs, rendering it more suitable for industrial applications. In parallel, modulating the relative expression of functionally interconnected genes has been widely adopted as a core strategy in metabolic engineering. As pivotal regulatory elements governing gene expression, RBSs exhibit convenient operability, tunable translational strength and negligible metabolic burden on chassis strains, making them ideal primary targets for regulatory optimization. Wei et al. optimized the RBS sequences of glycerol utilization pathway genes *glpF*, *dhaD*, and *dhaK* individually, which endowed *C. glutamicum* with the capacity to utilize glycerol (Wei et al. 2022). Through the strategy of RBS library construction, Shi et al. reconstructed xylose isomerase and Weimberg pathways in *C. glutamicum*, enabling high-efficiency co-utilization of glucose and xylose. This work establishes an efficient platform for low-cost and sustainable chondroitin bioproduction (Shi et al. 2025). Therefore, fine-tuning key heparosan biosynthetic genes through RBS engineering may act as an effective strategy to improve heparosan titer.

In this study, we systematically engineered *C. glutamicum* to achieve heparosan production through two complementary strategies: (i) genome-scale modulation of UDP-sugar precursors biosynthesis, and (ii) translation-level fine-tuning of the *kfiB-kfiC-kfiA* expression cassette via a random RBS library. By integrating these approaches, we aimed to engineer a genetically stable *C. glutamicum* chassis for heparosan synthesis, providing a viable platform for the efficient and safe production of heparosan and its derivatives.

## Materials and methods

### Strains and reagents

All strains used in this study are listed in Suppl. Table 1. *E. coli* DH5α was used for plasmid construction and preservation, while *C. glutamicum* ATCC 13032 and its derivative strains were used for heparosan biosynthesis.

Plasmid extraction kits and DNA gel extraction kits were obtained from Sangon Biotech (Shanghai, China). Phanta Flash Master Mix (Vazyme, Nanjing, China) was used for polymerase chain reaction (PCR) amplification of key fragments and Rapid Taq Master Mix (Vazyme, Nanjing, China) was used for colony PCR. Seamless cloning kits were purchased from Beyotime Biotechnology (Shanghai, China). Unless otherwise stated, all reagents used in this study were obtained from Sangon Biotech (Shanghai, China).

### Construction of recombinant plasmids

The pXMJ19 plasmid, which carries an IPTG-inducible promoter, was employed as the expression backbone for key genes. All plasmids used in this study were presented in Suppl. Table 2, with relevant primers listed in Suppl. Table 3. All genetic manipulations for plasmid construction were performed according to standard protocols.

Heparosan biosynthetic genes *kfiA*, *kfiC*, and the complex-stabilizing protein encoding gene *kfiB* were derived from *E. coli* K5. The UDP-GlcA and UDP-GlcNAc biosynthetic pathway genes, including *glmM*, *ugD*, *glmU*, *glmS*, *galU* and the corresponding homologous arm sequences, were all cloned from the genome of *C. glutamicum* ATCC 13032. During the construction of knockout and overexpression plasmids, the target genes *galE*, *pyrG*, *pta*, *ackA*, *pyrH*, *ndk* and their homologous arm sequences were all amplified from the *C. glutamicum* ATCC 13032 genome.

### Media and culture conditions

LB medium (5 g/L yeast extract, 10 g/L tryptone, 10 g/L NaCl, and 20 g/L agar when needed) was used for routine cultivation and plate screening of *E. coli*. LBB medium (5 g/L yeast extract, 10 g/L tryptone, 18.5 g/L brain heart infusion, 5 g/L NaCl, and 20 g/L agar when needed) was used for routine cultivation and plate screening of *C. glutamicum*. Competent medium (5 g/L yeast extract, 10 g/L tryptone, 18.5 g/L brain heart infusion, 5 g/L NaCl, 30 g/L L-glycine, 4 g/L isoniazid, and 1 mL/L Tween 80) was used for preparing competent cells and LBHIS medium (5 g/L yeast extract, 5 g/L tryptone, 2.5 g/L NaCl, 18.5 g/L brain heart infusion, and 91 g/L sorbitol) was used for the recovery incubation of *C. glutamicum* after electroporation. All *E. coli* strains were cultivated at 37℃ with shaking at 220 rpm for 12–16 h or under static conditions overnight. Kanamycin (50 mg/L) and chloramphenicol (34 mg/L) were supplemented when necessary. All recombinant *C. glutamicum* strains were cultivated at 30℃ with shaking at 220 rpm for 14–18 h or under static conditions for 48 h. Kanamycin (25 mg/L) and chloramphenicol (17 mg/L) were supplemented when necessary.

For shake-flask fermentation, all recombinant strains were pre-cultured in seed medium (20 g/L corn steep powder, 1 g/L KH_2_PO_4_, 0.5 g/L (NH_4_)_2_SO_4_, 1.25 g/L urea, 25 g/L glucose, pH adjusted to 7.0 with KOH) at 30℃, 220 rpm for 18–22 h. The seed culture was then inoculated into fermentation medium (15 g/L corn steep powder, 1 g/L KH_2_PO_4_, 40 g/L (NH_4_)_2_SO_4_, 0.18 g/L Fe_2_(SO_4_)_3_, 100 g/L glucose, 1 g/L MgSO_4_, pH adjusted to 7.0 with KOH) to achieve an initial OD_600_ of 1.6, followed by cultivation at 30℃, 220 rpm for 72 h. Chloramphenicol (17 mg/L) was added to the seed and fermentation media to maintain plasmid stability, and 0.5 mM isopropyl β-D-1-thiogalactopyranoside (IPTG) was added 2 h after fermentation initiation.

For fed-batch fermentation, the seed culture was incubated for approximately 20 h, and a 5% inoculation ratio was used to transfer the seed into the fermentation medium. The fed-batch fermentation medium contained: 15 g/L corn steep powder, 1 g/L KH_2_PO_4_, 40 g/L (NH_4_)_2_SO_4_, 0.18 g/L Fe_2_(SO_4_)_3_, 50 g/L glucose, 1 g/L MgSO_4_, pH adjusted to 7.0 with KOH. During fermentation, pH was controlled at 7.0 with 2 M KOH, a 500 g/L glucose solution was used as the fed-batch carbon source, and 10% antifoam was added as required.

### Genome editing of *C. glutamicum*

Genome editing of *C. glutamicum*, including gene knockout, and insertion of a strong promoter (P_*tac*_) or RBS, was performed using plasmid pK18mobsacB (Schäfer et al. 1994). First, the upstream and downstream homologous sequences flanking the target region, each approximately 1000 bp in length, were amplified from the *C. glutamicum* genome by PCR. The enhancer elements (P_*tac*_ and RBS) were introduced via primer sequences to construct the corresponding gene editing vectors. Notably, only the core region of the P_*tac*_ promoter (excluding the inducible regulatory region) was inserted upstream of each target gene to drive constitutive overexpression. Furthermore, to minimize confounding experimental factors, we retained the endogenous RBS sequence of each target gene without deletion or substitution during insertion of the P_*tac*_ promoter. Subsequently, the corresponding plasmids were electroporated into *C. glutamicum* competent cells and after 2–3 h of incubation, the mixtures were spread on antibiotic-containing medium and incubated statically for 48 h at 30℃. Then, all single colonies were picked and streaked onto antibiotic-free plates containing 100 g/L sucrose to eliminate the plasmids. Finally, positive clones were screened and verified by colony PCR and DNA sequencing.

### Growth curve determination of recombinant *C. glutamicum*

To investigate the effect of genome editing on the growth of recombinant strains, growth curves were monitored. Briefly, a single colony was picked and activated in 4 mL of LBB liquid medium at 30℃, 220 rpm for 14–18 h. Then, the activated culture was inoculated into a 250 mL shake flask containing 50 mL of fresh LBB liquid medium at a 1% inoculation ratio and cultivated at 30℃, 220 rpm. Samples were collected every 2 h post-inoculation to measure the optical density at 600 nm (OD_600_) until all recombinant strains reached the stationary phase.

### Biomass measurement and cell morphology analysis in fed-batch fermentation

Cell growth was monitored via OD_600_ measurement with a UV-visible spectrophotometer. For dry cell weight (DCW) determination, 10 mL of culture broth was collected by centrifugation. The cell pellet was washed three times with deionized water and subsequently dried to constant weight at 65℃. After cooling to room temperature, the DCW was measured gravimetrically. Fermentative residual glucose was determined with glucose test strips. Morphological changes of cells at different fermentation stages were observed after crystal violet staining using an Olympus CX33 microscope (Olympus, Japan), equipped with a WHSZ15X-H eyepiece (10 ×) and a CXPL100XO oil immersion objective (100 ×). Micrographs were captured with LIOO (LIOO software, Germany), and the built-in calibration function was used to calibrate the scale bar for all cell images.

### Isolation and purification of heparosan

Heparosan was isolated and purified as reported previously (Hu et al. 2022a; Yu et al. 2025). First of all, 30–50 mL of fermentation broth was collected and centrifuged at 7000 rpm for 10 min to harvest the cell pellet. The pellet was then resuspended and washed three times with deionized water to remove medium impurities. Following washing, the cells were resuspended in deionized water and disrupted via high-pressure homogenization at 1000 bar for 4 min. The treated mixture was heated at 65℃ for 2 h to denature proteins and then centrifuged at 12,000 rpm for 10 min to collect the supernatant. The supernatant was mixed with three volumes of ethanol, followed by incubation at 4 °C overnight. Afterwards, the mixture was centrifuged at 12,000 rpm for 10 min to collect the insoluble precipitate. The resulting precipitate was dried at 65℃, redissolved in deionized water, and the ethanol precipitation was repeated three times to minimize interference from intracellular small-molecule carbohydrate analogs.

### Characterization, quantification and molecular weight determination of heparosan

Heparosan produced in this study was depolymerized by heparin lyase III at 37℃, 100 rpm for 24 h, and the generated disaccharides were then analyzed using a Waters Quattro XE mass spectrometer. The target unsaturated disaccharide ΔUA-GlcNAc displays a characteristic peak at m/z = 378.10. To quantify heparosan, the modified carbazole assay was carried out (Bitter and Muir 1962), using D-glucuronic acid as an external standard. Briefly, 500 µL of test sample solution was mixed with 3 mL of H_2_SO_4_ and heated at 100℃ for 20 min. After cooling to room temperature, 100 µL of carbazole reagent was added and the mixture was heated at 100℃ for 15 min. After cooling to room temperature, the absorbance was measured at 530 nm, and the heparosan titer was calculated based on the D-glucuronic acid content.

The molecular weight distribution of heparosan was determined by high-performance gel permeation chromatography (HPGPC, Waters 1515). Samples were dissolved in deionized water and filtered through a 0.22 μm membrane. The analysis was performed using a refractive index detector, with 0.05 M NaCl solution serving as the mobile phase at a constant column temperature of 40℃ and a flow rate of 0.50 mL/min. Pullulan standards with known molecular weights were used for calibration. The weight-average molecular weight (M_w_), number-average molecular weight (M_n_), and polydispersity index (PDI, M_w_/M_n_) were calculated based on the established standard curve.

### Construction of a random RBS library in *C. glutamicum*

To visualize the strength of different RBS sequences, a reporter plasmid pXMJ19-M was created by inserting the red fluorescent protein coding gene (*mCherry*) into pXMJ19. According to existing studies, AGGAG was chosen as the conserved Shine-Dalgarno (SD) sequence (Wei et al. 2022; Zhang et al. 2015), and three primer pairs (RBS-wk1-f/r, RBS-wk2-f/r and RBS-wk3-f/r) containing 6–8 random bases (AGGAG(N)_6–8_) were designed to introduce randomness. Following PCR amplification, the reaction mixture was directly transformed into *E. coli* DH5α to construct a plasmid library harboring various RBS variants. Subsequently, all clones were inoculated into LB liquid medium and cultivated overnight in 96-well plates for plasmid extraction. Then, a mixture of plasmids with high diversity was transformed into *C. glutamicum* for subsequent screening. A total of 384 clones were picked and inoculated into 96-well plates containing LBB liquid medium and cultivated at 30℃, 220 rpm for 24 h, with IPTG added at a final concentration of 0.5 mM at 2 h of cultivation. After fluorescence intensity measurement, strains with distinct fluorescence intensities were selected again and inoculated into shake flasks for further measurement and sequence verification.

### Measurement of fluorescence intensity

Red fluorescence intensity was measured with excitation and emission wavelengths of 580 nm and 610 nm using a microplate reader, and the gain was fixed at 100 for all measurements. Fluorescence values of experimental groups were corrected by subtracting the background signal of the empty-vector control strain. Relative fluorescence intensity was determined by normalizing the fluorescence intensity to the cell density (OD_600_).

### Statistical analysis

Data analysis was conducted using GraphPad Prism 10 (GraphPad Software, USA). Unless otherwise stated, all experiments were performed with three independent biological replicates. All results are expressed as the mean ± standard deviation, and the average value of each biological replicate was taken as the statistical unit for analysis. A two-tailed Student’s t-test was used for comparisons between two groups. For datasets containing multiple experimental groups, Welch’s one-way ANOVA followed by Dunnett’s T3 post-hoc test was performed.

## Results

### Construction of the heparosan biosynthetic pathway in *C. glutamicum*

**Fig. 1:**
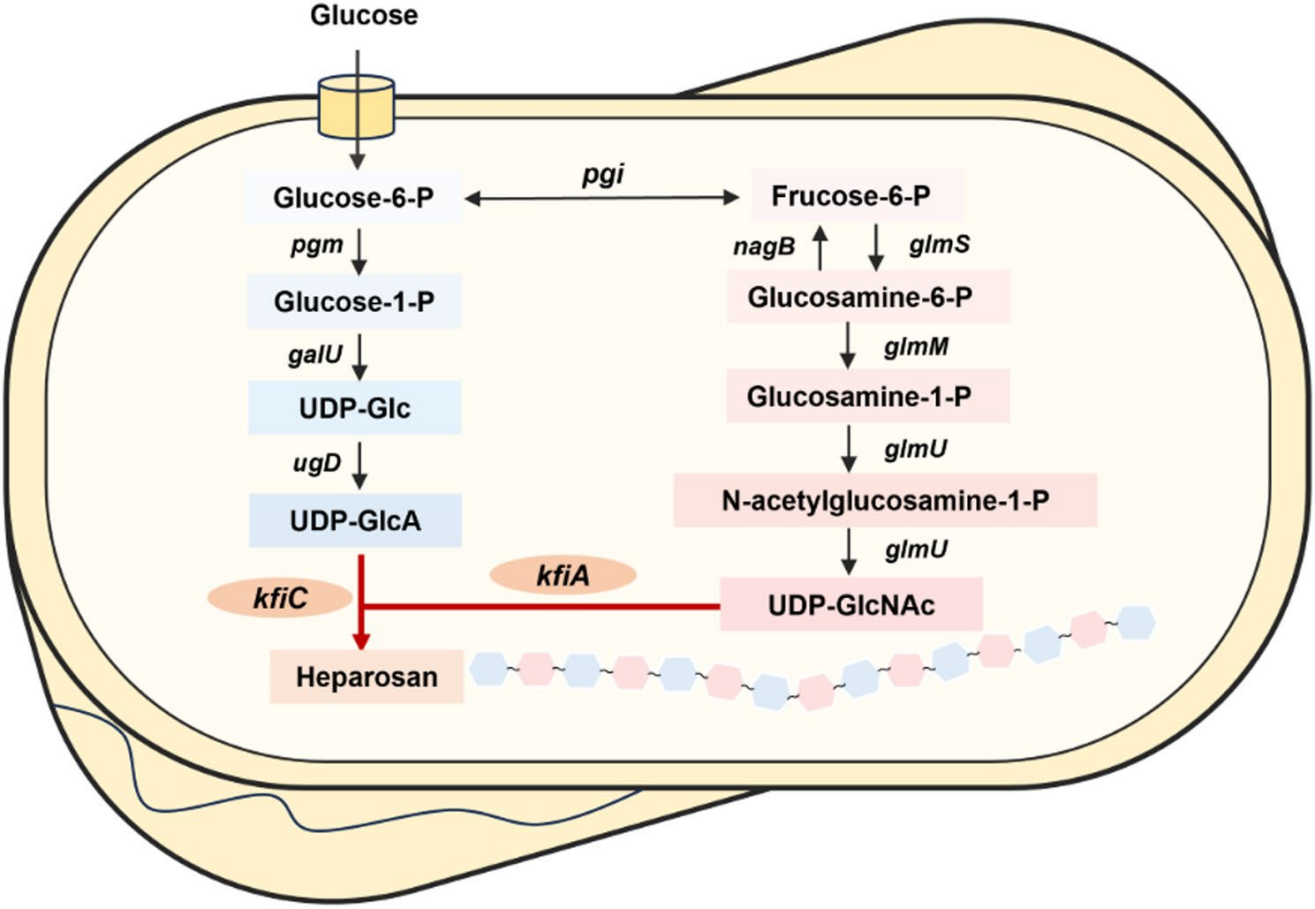
Heparosan biosynthetic pathway in recombinant *C. glutamicum*. The black arrows represent the endogenous genes and the red thick arrows represent two essential heterologous genes, *kfiA* (encoding N-acetylglucosaminyltransferase) and *kfiC* (encoding glucuronosyltransferase), which are derived from *E. coli* K5 and participate in the elongation of the heparosan chain. *pgi*, encoding glucose-6-phosphate isomerase; *pgm*, encoding phosphoglucomutase; *galU*, encoding glucose-1-phosphate uridylyltransferase; *ugD*, encoding UDP-glucose dehydrogenase; *nagB*, encoding glucosamine-6-phosphate deaminase; *glmS*, encoding L-glutamine-D-fructose-6-phosphate aminotransferase; *glmM*, encoding phosphoglucosamine mutase; *glmU*, encoding bifunctional UDP-N-acetylglucosamine pyrophosphorylase/glucosamine-1-phosphate N-acetyltransferase

**Fig. 2:**
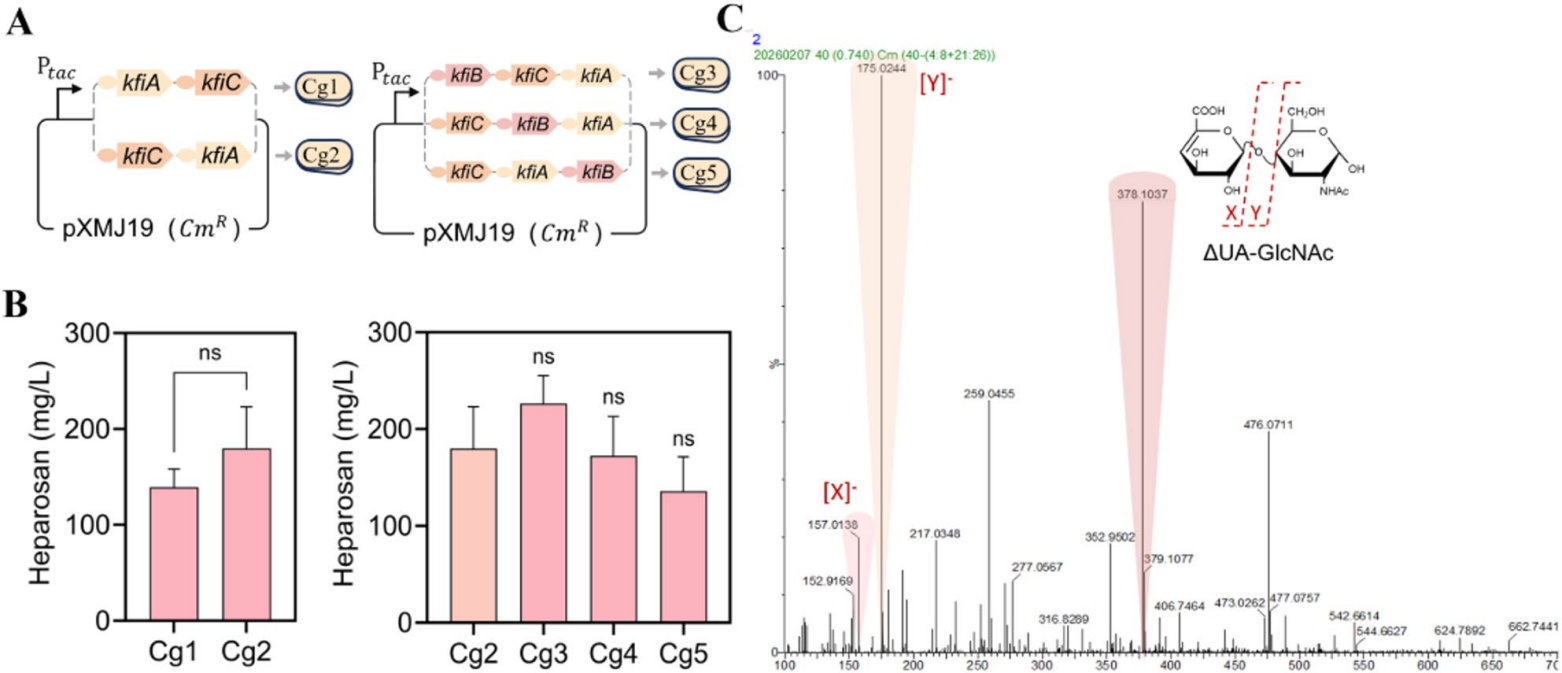
Construction of heparosan biosynthetic pathway in *C. glutamicum*. **A** Schematic diagram of different recombinant plasmids. Different colors are applied to distinguish the three distinct genes (*kfiA*, *kfiB*, and *kfiC*) as well as their native RBS elements (shown as ellipses). **B** Heparosan titer of recombinant strains harboring plasmids with distinct arrangements of genes *kfiA*, *kfiB*, and *kfiC*. **C** TOF-MS result of hydrolysate from Cg2 fermentation sample treated with heparinase III. ΔUA-GlcNAc: unsaturated disaccharide after cleavage (m/z = 378.10). Data are presented as the mean ± standard deviation from three independent biological replicates (*n* = 3). ns, no significant difference (*p* ≥ 0.05)

After analyzing the metabolic network of *C. glutamicum* ATCC 13032, we found that two key precursors, UDP-GlcA and UDP-GlcNAc, which are indispensable for heparosan biosynthesis, can be inherently produced (Fig. 1). However, the endogenous enzymes catalyzing the downstream conversion of UDP-GlcA and UDP-GlcNAc to heparosan are absent. Therefore, we first cloned and inserted the *E. coli* K5 glycosyltransferase coding genes, *kfiA* and *kfiC*, into pXMJ19 to construct the plasmids pXMJ19*-kfiA-kfiC* and pXMJ19-*kfiC-kfiA* (Fig. 2A), and then transformed them into Cg0 independently to generate strains Cg1 and Cg2. According to the results (Fig. 2B), Cg2 (179.86 mg/L) exhibits a slight production advantage over Cg1 (139.17 mg/L), indicating that different expression cassettes of key genes may affect the biosynthesis of the target product (Hu et al. 2024). This phenomenon can be attributed to the fact that varied expression cassettes change the relative expression intensity of the KfiA and KfiC, thereby interfering with the assembly of functional catalytic complexes KfiA-KfiC. After depolymerization with heparinase III, the unsaturated disaccharide ΔUA-GlcNAc (m/z = 378.10) was characterized, confirming successful heparosan synthesis in Cg2 (Fig. 2C).

Based on previous investigations, we hypothesized that KfiB, a functional protein that stabilizes the KfiA-KfiC complex and plays an important role in heparosan synthesis and transport in *E. coli* K5 (Leroux and Priem 2016; Zhang et al. 2012), might help increase the intracellular content of heparosan in *C. glutamicum*. To verify this speculation, three new plasmids, pXMJ19-*kfiB-kfiC-kfiA*, pXMJ19-*kfiC-kfiB-kfiA*, and pXMJ19-*kfiC-kfiA-kfiB* were constructed and transformed into Cg0 to generate recombinant strains Cg3, Cg4, and Cg5. As shown in Fig. 2B, recombinant strains Cg3, Cg4, and Cg5 produced heparosan at 226.37, 172.46, and 136.00 mg/L, respectively, suggesting that the *kfiB-kfiC-kfiA* cassette may possess higher potential for enhancing heparosan biosynthesis, although this difference was not statistically significant. This result suggests that balancing the expression of KfiA, KfiB, and KfiC may be a feasible strategy to enhance heparosan production.

### Increase the intracellular contents of UDP-GlcA and UDP-GlcNAc to improve heparosan production

**Fig. 3:**
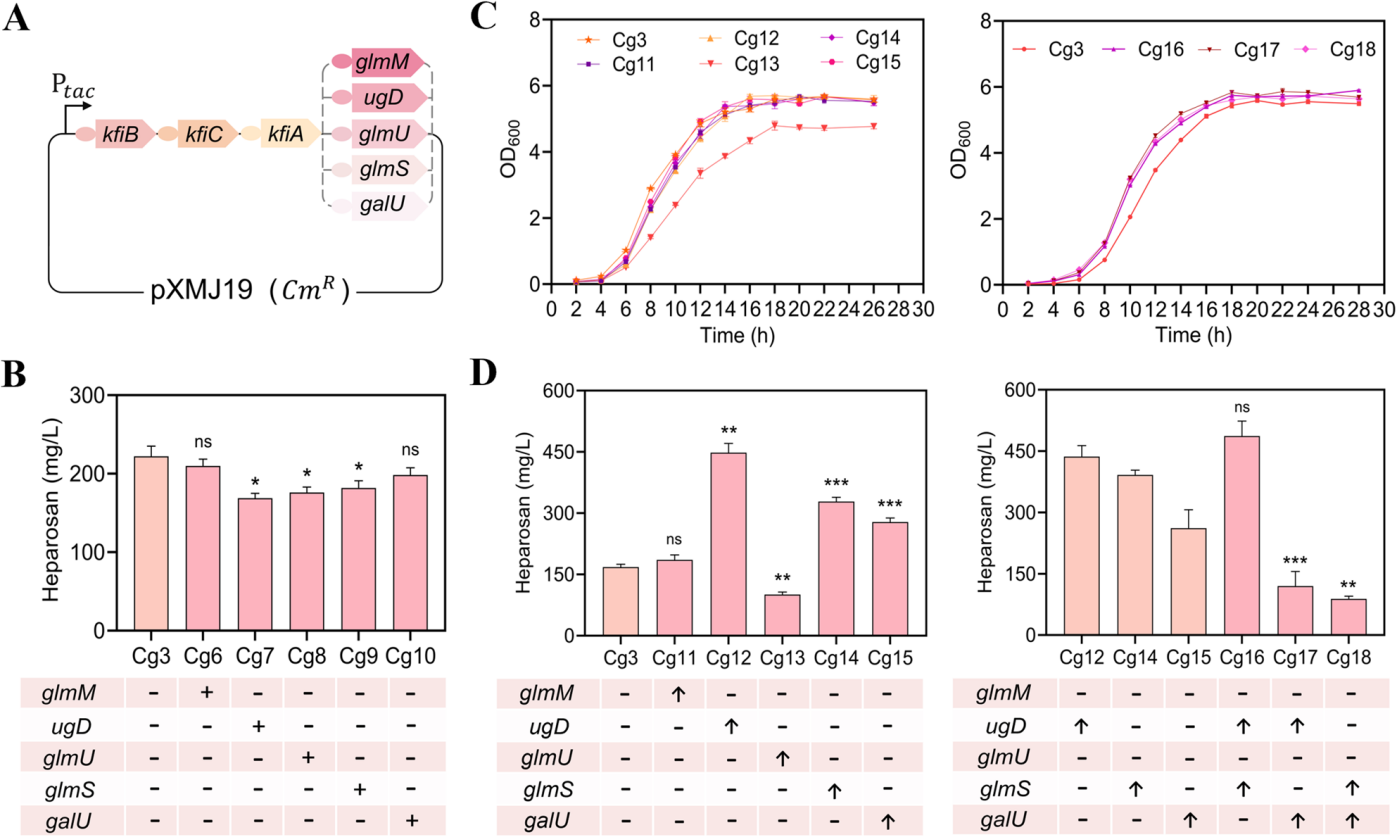
Promotion of UDP-GlcA and UDP-GlcNAc for improved heparosan titer. **A** Schematic diagram of different recombinant plasmids. Different colors are applied to distinguish different genes as well as their native RBS elements (shown as ellipses). **B** Heparosan titer of recombinant strains overexpressing *glmM*, *ugD*, *glmU*, *glmS*, or *galU* with plasmids. **C** Growth curves of recombinant strains with different genomic modifications. **D** Heparosan titer of recombinant strains with different genomic modifications. Data from Cg16–Cg18 were statistically compared against the control Cg12. The arrow represents genomic enhancement of the target gene by P_*tac*_ insertion. Data are presented as the mean ± standard deviation from three independent biological replicates (*n* = 3). *p < 0.05, **p < 0.01, ***p < 0.001; ns, no significant difference (*p* ≥ 0.05)

To further improve heparosan biosynthesis, five genes, *glmM*, *ugD*, *glmU*, *glmS*, and *galU*, which are indispensable in the biosynthesis of UDP-GlcA and UDP-GlcNAc (Hu et al. 2022a; Zhang et al. 2018), were cloned to form plasmids pXMJ19-*kfiB-kfiC-kfiA-X* (X=*glmM*, *ugD*, *glmU*, *glmS* or *galU*) (Fig. 3A). These plasmids were then transformed into Cg0 to generate Cg6–Cg10, respectively. Disappointingly, the introduction of these genes did not improve the heparosan titer (Fig. 3B), which may be partially attributed to the increased vector size and structural instability arising from the insertion of an extra gene into the existing three-gene cassette (Kitade et al. 2013).

To increase the intracellular content of UDP-GlcA and UDP-GlcNAc in a stable manner, we upregulated the expression of target genes at the genome level. Briefly, a strong promoter, P_*tac*_, was inserted upstream of *glmM*, *ugD*, *glmU*, *glmS*, or *galU* in Cg3 (Suppl. Fig. 1) to construct recombinant strains Cg11, Cg12, Cg13, Cg14, and Cg15. We first evaluated the growth profiles of these recombinant strains (Fig. 3C). Compared to the control, only Cg13 showed a slight decrease in growth performance. Furthermore, we observed that almost all recombinant strains synthesized heparosan more efficiently than Cg3, except Cg13 (Fig. 3D). Compared with Cg3, recombinant strains Cg12, Cg14, and Cg15 exhibited significantly improved heparosan titers of 447.97, 328.61, and 278.34 mg/L, respectively, which were 2.67, 1.96, and 1.66-fold that of the control. The three superior strains were then combined through pairwise combinations to construct recombinant strains Cg16, Cg17, and Cg18. The growth profiles (Fig. 3C) confirmed that this reinforcement strategy did not adversely affect bacterial growth. However, only Cg16 (486.72 mg/L) exhibited a slight improvement in heparosan biosynthesis (Fig. 3D), which may be related to carbon source redistribution in the recombinant strains.

### Strategic regulation of the metabolic pathway for efficient intracellular accumulation of UDP-GlcA and UDP-GlcNAc

Microbial physiological metabolism resembles a complex network involving the flux and conversion of many metabolites. Eliminating redundant metabolic branches and enhancing core pathways may help divert more carbon flux into the target biosynthetic pathway. For example, the double knockout of *arcA* and *iclR* not only reduced the accumulation of the toxic metabolite acetate but also significantly enhanced N-acetylneuraminic acid biosynthesis in *E. coli* (Qi et al. 2025). Knocking out *pfkA* in the EMP pathway reduced carbon source diversion, and redirected carbon flux toward chondroitin synthesis, significantly improving the chondroitin titer (Zhang et al. 2018).

**Fig. 4:**
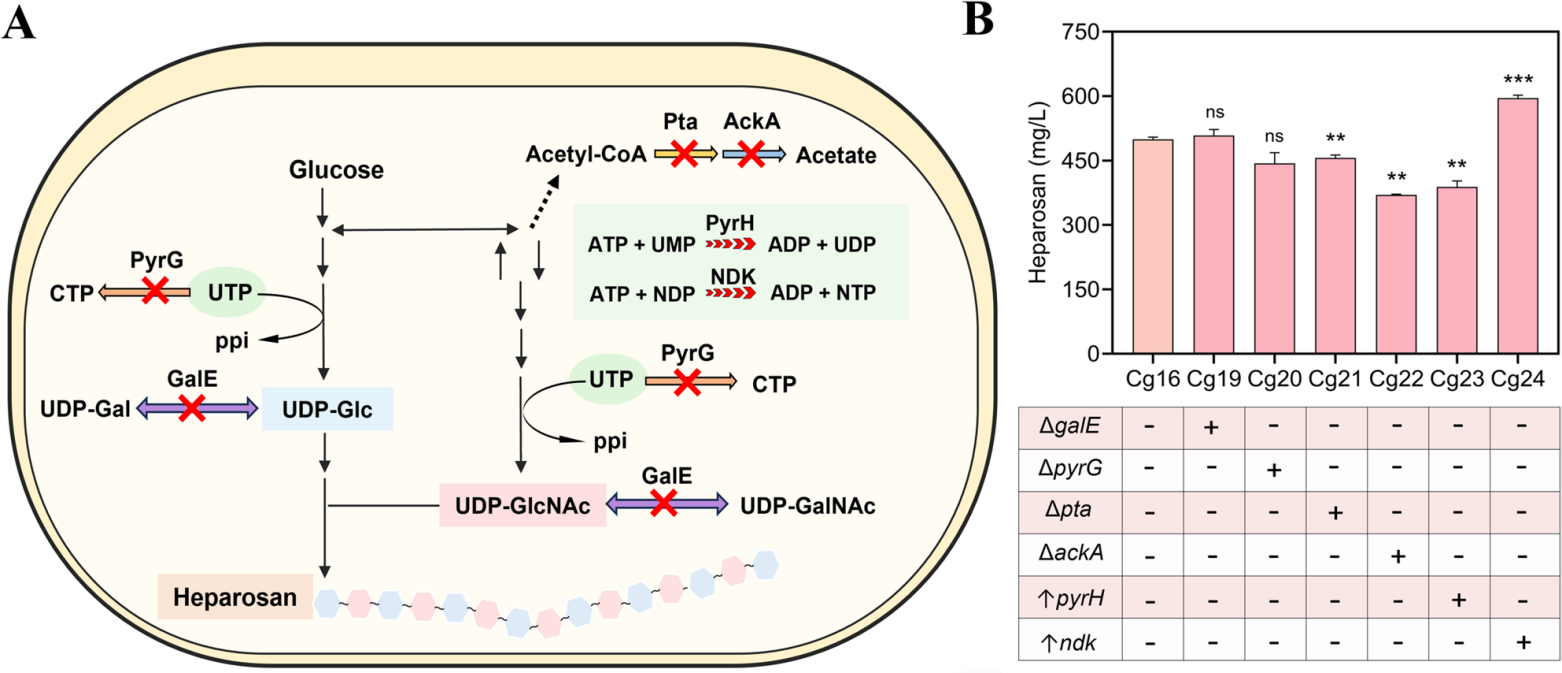
Strategic regulation of metabolic pathway in recombinant *C. glutamicum* for improved heparosan biosynthesis. **A** Schematic diagram of strategic regulation. The region highlighted by the green square represents pathways involved in UTP biosynthesis (N = A/U/C/G). The red cross indicates knockout, and the red arrow indicates enhanced expression with robust RBS. GalE: UDP-glucose 4-epimerase; PyrG: cytidine triphosphate synthase; Pta: phosphate acetyltransferase; AckA: acetate kinase; PyrH: uridylate kinase; NDK: nucleoside diphosphate kinase. **B** Heparosan titer of recombinant strains with different genomic modifications. Δ: gene knockout; ↑: gene upregulation with robust RBS. Data are presented as the mean ± standard deviation from three independent biological replicates (*n* = 3). **p < 0.01, ***p < 0.001; ns, no significant difference (*p* ≥ 0.05)

Based on the metabolic pathway of UDP-GlcA and UDP-GlcNAc in recombinant *C. glutamicum*, separate gene knockouts were performed for *galE*, *pyrG*, *ackA*, and *pta*, which appear to be involved in unnecessary branch pathways and consume intermediates in UDP-GlcA and UDP-GlcNAc biosynthesis (Fig. 4A). Since the synthesis of UDP-GlcA and UDP-GlcNAc demands large amounts of UTP, the intracellular availability of UTP likely serves as a critical rate-limiting factor (Deng et al. 2025). Therefore, *pyrH* that encodes the enzyme catalyzing the conversion of uridine monophosphate (UMP) to uridine diphosphate (UDP) and *ndk* that encodes the enzyme catalyzing the conversion of uridine diphosphate (UDP) to uridine triphosphate (UTP), which may help improve UTP synthesis (Fig. 4A), were enhanced by replacing their native RBS sequences with the robust RBS motif (AGGAGTGCCCATA). In total, six recombinant strains were obtained. As shown in Fig. 4B, the heparosan titers of recombinant strains Cg19, Cg20, Cg21, and Cg22 did not improve as expected, suggesting that the disruption of these pathways was not a reasonable strategy. However, compared with the control strain Cg16, *pyrH* overexpression resulted in a lower heparosan titer, while *ndk* overexpression enhanced the titer by 19.20%, suggesting that the conversion of UDP to UTP, rather than UMP to UDP, constitutes a key limiting factor for UTP supply.

### Fine-tuning of individual genes within the *kfiB*-*kfiC*-*kfiA* expression cassette for increased heparosan titer

1. Construction of the RBS sequence library

The ribosome binding site is a functional element responsible for ribosome recognition and recruitment, thereby initiating translation of the target gene. An RBS can be divided into the SD sequence and the spacer sequence. The SD sequence is highly conserved (typically 5’–AGGAG–3’ in *C. glutamicum*) and mediates ribosome binding, while the spacer sequence is inherently variable and closely associated with ribosome binding efficiency (Wei et al. 2022; Zhang et al. 2015). By screening suitable RBS sequences, the expression of key genes can be effectively regulated (Shi et al. 2025). Qi et al. constructed a series of RBS mutants with varying strengths for *glmS*_A_*, *glmM*, and *glmU*. Using an efficient screening strategy, they obtained the optimal RBS combination for N-acetylneuraminic acid synthesis in *E. coli* (Qi et al. 2025). Zhang et al. combined different RBS sequences with specific genes involved in UDP-GlcA and UDP-GlcNAc synthesis, which significantly improved the UDP-GlcNAc/UDP-GlcA ratio and made it more suitable for chondroitin production (Zhang et al. 2018).

**Fig. 5:**
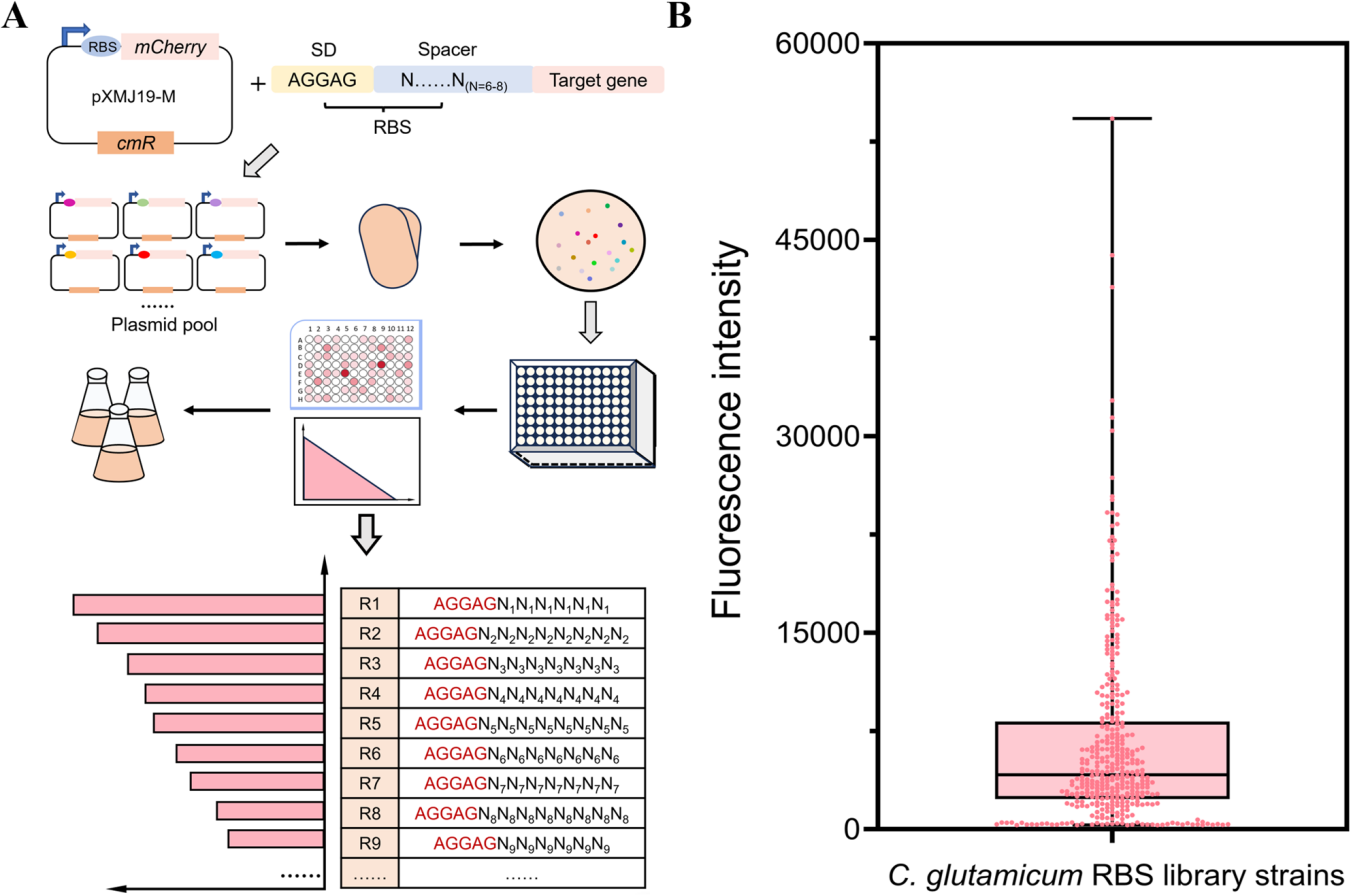
Construction of the RBS random sequence library. **A** Procedures of construction and screening of RBS library. Primers carrying random sequences were applied to amplify the initial reporter vector (pXMJ19-M) to generate a random library of RBS mutants. **B** The fluorescence intensity distribution of 384 individual colonies picked from the RBS library. The box represents the 25–75% quantile range, the horizontal line inside the box indicates the median, and dots correspond to fluorescence intensity of all individual strains

**Fig. 6:**
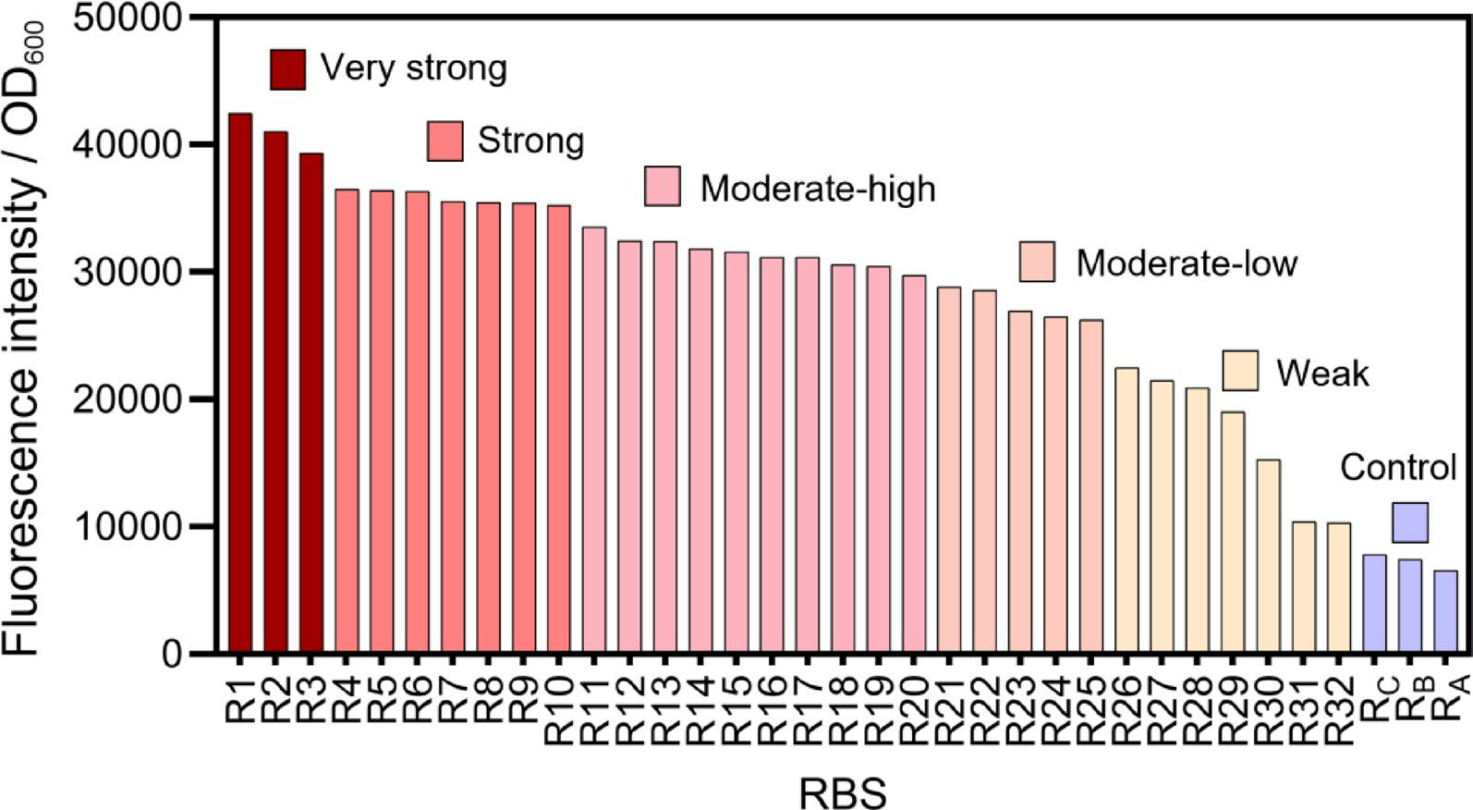
Relative fluorescence intensity of different RBS sequences on *mCherry* expression. R1–R32 represent different RBSs derived from the RBS library we constructed, and these RBS variants were divided into five groups with different translational strengths according to the relative fluorescence intensities: very strong, strong, moderate-high, moderate-low and weak. The color gradient from dark to light represents the gradual reduction in RBS strength. R_B_, R_C_ and R_A_ represent the RBSs of *kfiB*, *kfiC*, and *kfiA* in plasmid pXMJ19*-kfiB-kfiC-kfiA*, respectively

In this work, we used *mCherry* as a reporter gene to visualize translational efficiency. A seeding sequence, AGGAG(N)_6–8_, which covers 10^4^–10^5^ possible sequences, was designed and used for construction of the RBS library (Fig. 5A). As shown in Fig. 5B and Suppl. Fig. 2, the red fluorescence intensities of 384 distinct *C. glutamicum* colonies ranged from 319 to 54,269, indicating that the RBS library we constructed has a broad spectrum of gene expression regulation efficiency. Subsequently, 40 single colonies corresponding to diverse RBS strengths were picked for measuring relative fluorescence intensities. Meanwhile, the specific RBS sequences of these colonies were determined and deduplicated. These RBS sequences were then classified into five categories according to their translation strengths and summarized in Fig. 6 and Suppl. Table 4.

2. Precise regulation of *kfiB*, *kfiC*, and *kfiA* expression for increased heparosan titer

The above results indicate that regulating the relative expression strengths of key genes could represent an effective strategy for enhancing heparosan biosynthesis. To achieve fine-tuned expression of *kfiB*, *kfiC*, and *kfiA*, we first evaluated the existing RBS intensities of these genes in plasmid pXMJ19-*kfiB-kfiC-kfiA*, as shown in Fig. 6. The results showed that the native RBS strengths of these three genes were relatively low, which may be a limiting factor in the further improvement of product titer. On this basis, the RBS sequences obtained from the RBS library were exploited and combined with *kfiB*, *kfiC*, and *kfiA* using two different strategies: (i) improving the expression of all three genes with RBSs of the same strength. (ii) upregulating one of them while maintaining the others at moderate levels. In total, 15 recombinant plasmids, p19BCA-rbsX (X = 1, 2 …15), were constructed, and 13 recombinant strains were obtained after transformation into *C. glutamicum*.

**Fig. 7:**
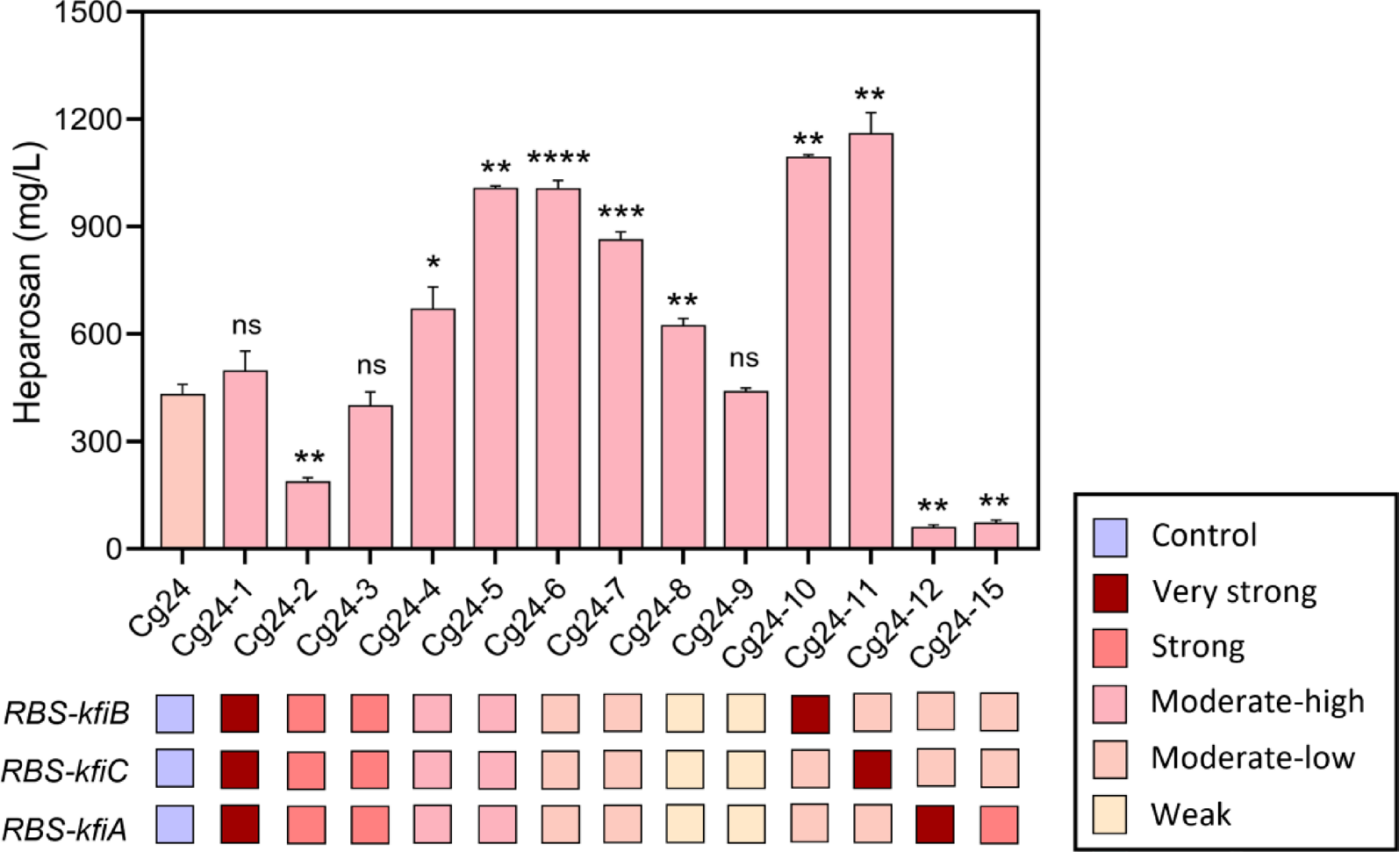
Heparosan titer of recombinant strains with different p19BCA-rbsX plasmids. The color blocks below represent the RBS strengths of three genes (*kfiB*, *kfiC*, and *kfiA*) in each recombinant plasmid. Data are presented as the mean ± standard deviation from three independent biological replicates (*n* = 3). *p < 0.05, **p < 0.01, ***p < 0.001, ****p < 0.0001; ns, no significant difference (*p* ≥ 0.05)

As shown in Fig. 7, when the expression of *kfiB*, *kfiC*, and *kfiA* were enhanced equally, the heparosan titer showed an increase-then-decrease trend with increasing RBS strength. The highest heparosan titers were achieved when the key genes were expressed at moderate-high (1008.21 mg/L) or moderate-low (1007.28 mg/L) levels, indicating that appropriate gene expression levels are more conducive to product synthesis. Further analysis demonstrated that by maintaining moderate expression of two key genes while enhancing the other, recombinant strains Cg24-10, Cg24-11, Cg24-12, and Cg24-15 exhibited significant differences in heparosan production. Individual upregulation of *kfiB* and *kfiC* resulted in heparosan titers of 1095.27 mg/L (Cg24-10) and 1161.37 mg/L (Cg24-11), respectively, with the latter representing the highest titer among all recombinant strains, which was 168.92% higher than that of Cg24. However, when *kfiA* was upregulated, recombinant strains Cg24-12 and Cg24-15 almost lost the ability to synthesize heparosan. This suggests that the expression level of *kfiC* may be relatively insufficient, and upregulating its expression could facilitate the assembly of more KfiA-KfiC catalytic complexes (Hu et al. 2024; Leroux and Priem 2016). In contrast, strengthening *kfiA* might aggravate the imbalance between the two enzymes, resulting in a significant decrease in heparosan production.

### Scale-up production of heparosan in a 5 L bioreactor

To evaluate the heparosan synthesis potential of Cg24-11, fed-batch fermentation in a 5 L bioreactor was carried out. A cell growth-product synthesis two-stage fermentation strategy was employed. In the first stage, cell growth was maintained by controlling dissolved oxygen (DO) above 30% and residual glucose no less than 10 g/L. Meanwhile, to avoid cell damage caused by shear stress, DO was regulated by gradually increasing the agitation speed. When the cells entered the mid-to-late exponential growth phase, recombinant protein expression was induced to initiate product synthesis. Concurrently, DO was controlled at 10–30%, and a restricted feeding strategy was adopted by maintaining the residual sugar concentration at 1–5 g/L to weaken cell growth, thereby directing more carbon source toward heparosan biosynthesis.

**Fig. 8:**
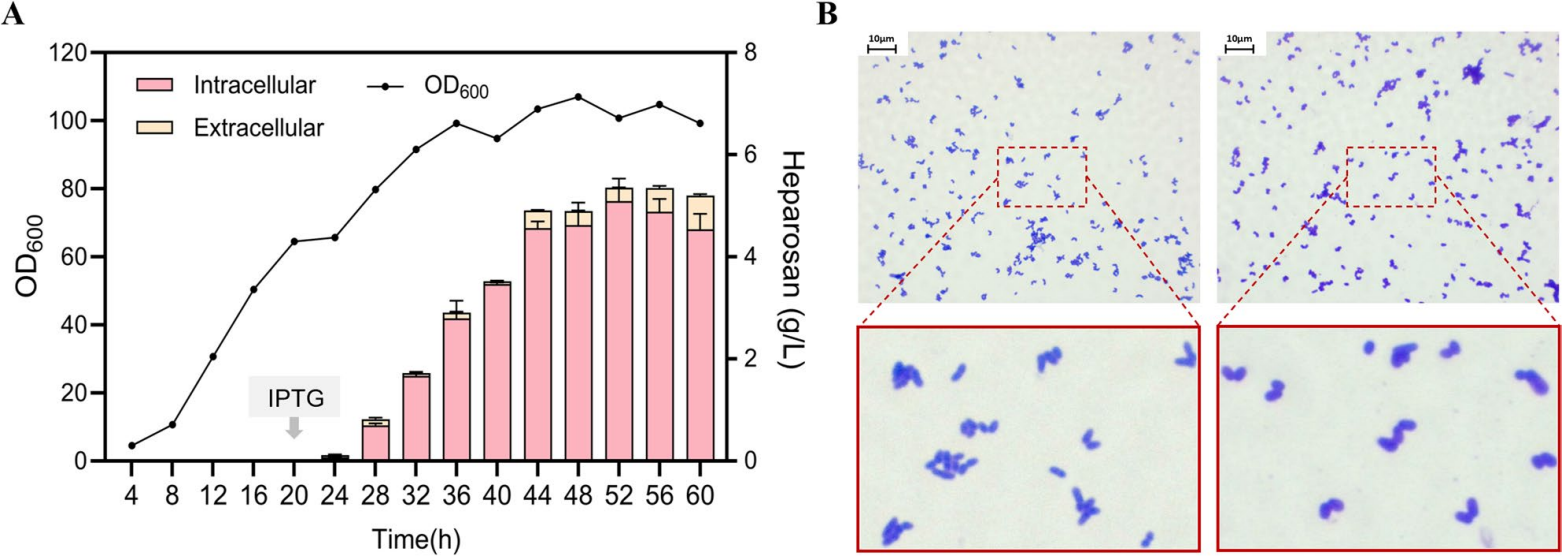
Fed-batch fermentation of recombinant strain Cg24-11 in a 5 L bioreactor. **A** Fed-batch fermentation of recombinant strain Cg24-11 for heparosan production. IPTG was added at 20 h of fermentation to trigger recombinant protein expression and product biosynthesis. The pink bar indicates intracellular heparosan titer, and the flesh-colored bar indicates extracellular heparosan titer at different times. The dark curve represents OD_600_ during fermentation. **B** Morphological observation during fermentation. Left: before induction; right: late fermentation stage after induction. Cells were observed under an optical microscope at 1000× magnification. Scale bar, 10 μm

As shown in Fig. 8, after IPTG addition (20 h), heparosan began to accumulate rapidly. At 52 h, the heparosan titer reached a maximum of 5.36 g/L, of which the intracellular product accounted for 95.08%, and the specific productivity at this time point was 0.14 g/g DCW. Furthermore, extracellular product content increased markedly starting at 44 h, indicating a certain degree of cell autolysis and release of intracellular heparosan. The apparent drop in measured heparosan titer at 60 h likely resulted from a combination of arrested product synthesis due to nutrient exhaustion, intracellular heparosan release triggered by cell lysis, and measurement deviations arising from continuous feeding in the late fermentation stage. The M_w_ and M_n_ of the sample were 208.42 kDa and 147.63 kDa, respectively, with a PDI of 1.41 (Suppl. Fig. 3). Morphological analysis showed that before induction, cells exhibited the typical rod-shaped morphology of *C. glutamicum*. As fermentation time extended, the transverse dimension of the rod-shaped cells gradually widened, and a morphological shift from rod to sphere was observed, indicating effective intracellular accumulation of heparosan.

## Discussion

In the present study, the biosafe bacterium *C. glutamicum* was developed as an efficient microbial cell factory to produce heparosan. Initially, we established the fundamental heparosan biosynthetic pathway in *C. glutamicum* via optimization of the expression frameworks for *kfiA*, *kfiB*, and *kfiC*. The results (Fig. 2B) demonstrated that the relative expression strengths of distinct functionally associated genes may influence the synthesis of the target product (Hu et al. 2024), providing a basis for subsequent engineering.

To promote intracellular accumulation of UDP-GlcA and UDP-GlcNAc, genes *glmM*, *ugD*, *glmU*, *glmS*, and *galU*, were individually overexpressed using plasmids. However, the corresponding recombinant strains failed to improve the heparosan titer (Fig. 3B), likely due to the inherent instability of the oversized vector. To steadily upregulate the expression of these genes, we modified the relevant genes at the genomic level by inserting a strong promoter, P_*tac*_, upstream of them. As shown in Fig. 3D, recombinant strains Cg11, Cg12, Cg14, and Cg15 all exhibited increased heparosan titers, among which Cg12, Cg14, and Cg15 showed more significant enhancement, reaching 2.67, 1.96, and 1.66-fold that of the control, respectively. However, we also observed that both cell growth and product synthesis in Cg13 were negatively affected, indicating that *glmU* overexpression resulted in simultaneous reductions in both biomass and product accumulation, consistent with the previous report (Hu et al. 2022b). This may be attributed to two independent potential causes associated with *glmU* overexpression. First, the bifunctional enzyme GlmU exhibits distinct catalytic efficiencies for its two consecutive reactions. Overexpression of *glmU* further amplifies this inherent catalytic imbalance, leading to substantial accumulation of the first-step product GlcNAc-1-P (Mengin-Lecreulx and van Heijenoort 1994). Therefore, excessive intracellular GlcNAc-1-P disrupts normal metabolic homeostasis, hinders downstream sugar nucleotide biosynthesis, and ultimately limits heparosan production. Second, *glmU* overexpression may also trigger intracellular accumulation of UDP-GlcNAc, which could impose feedback suppression on upstream pathways and disrupt the balanced ratio of two activated sugar precursors, thereby limiting the synthesis of heparosan. Finally, the recombinant strain Cg16 with both *ugD* and *glmS* overexpressed achieved a heparosan titer of 486.72 mg/L. Based on this result, we speculate that the conversion of UDP-Glc to UDP-GlcA and Fru-6-P to GlcN-6-P are the key rate-limiting steps for the synthesis of UDP-GlcA and UDP-GlcNAc (Hu et al. 2022a). Simultaneous overexpression of these two key genes may help elevate intracellular pools of essential activated sugar precursors, which could exert a relatively prominent stimulatory effect on heparosan biosynthesis. Furthermore, *pyrH* and *ndk* were individually upregulated at the genomic level by inserting a strong RBS. The results (Fig. 4B) showed that *ndk* overexpression (Cg24) promoted heparosan biosynthesis, while *pyrH* overexpression (Cg23) resulted in decreased heparosan production, suggesting that the conversion of UDP to UTP, instead of UMP to UDP, may constitute a rate-limiting step for UTP synthesis. Overexpression of *ndk* appears to facilitate the conversion of UDP to UTP and elevate intracellular levels of UTP, which may in turn contribute to the biosynthesis of UDP-GlcA and UDP-GlcNAc, thereby providing sufficient activated sugar precursors for heparosan synthesis. Through the above strategies, we constructed an *C. glutamicum* chassis with significantly enhanced heparosan production (from 226.37 mg/L to 595.39 mg/L). Considering the universality of UDP-precursors in glycosaminoglycan synthesis, this chassis is expected to serve as an efficient parent strain for the biosynthesis of other glycosaminoglycans.

Inspired by the results in Fig. 2B, we further fine-tuned the relative expression levels of *kfiA*, *kfiB*, and *kfiC* to investigate their effects on product biosynthesis. By constructing a random RBS library, we implemented two separate strategies to regulate the expression of these key genes. The results (Fig. 7) indicated that when the expressions of the target genes were maintained at an appropriate level, heparosan production was optimal. Both excessively high and low expression of the three genes led to decreased heparosan production. This phenomenon likely arises because heparosan biosynthesis relies on synergistic cooperation between KfiA and KfiC. Meanwhile, KfiB acts as an essential accessory protein responsible for sustaining the structural stability of the KfiA-KfiC complex; its intracellular abundance also exerts an evident facilitating effect on the assembly of functional heterodimers. RBS sequences serve as core translational regulatory elements that directly govern the translation initiation efficiency and resultant intracellular protein abundance of their downstream genes. Accordingly, we tuned the translational strength of RBS sequences upstream of these three genes to adjust the cellular pool of KfiA-KfiC catalytic heterodimers and ultimately realize adjustable heparosan titers. Further analysis indicated that the relative expression levels of *kfiA* and *kfiC* may play a critical role in heparosan biosynthesis. Upregulation of *kfiC* resulted in a heparosan titer of 1161.37 mg/L (Cg24-11), representing a 168.92% increase compared with the control. However, when *kfiA* was upregulated, strains Cg24-12 and Cg24-15 nearly lost the ability to produce heparosan. A plausible explanation is that an appropriate intracellular abundance of both proteins may help promote the formation of a more stable active heterodimer. Therefore, separate overexpression of *kfiA* and *kfiC* led to opposite effects on heparosan production. Moreover, future efforts could integrate the optimized *kfiB-kfiC-kfiA* expression cassette into the chromosome to create genetically stable recombinant *C. glutamicum*, providing reliable engineered strains for industrial applications.

To further evaluate whether the engineered strain Cg24-11 has potential for scaled-up heparosan production, fed-batch fermentation was carried out in a 5 L bioreactor. A growth-production stage-separated regulation strategy was implemented by modulating DO and residual sugar levels across different fermentation stages. Consequently, our strain yielded a peak heparosan titer of 5.36 g/L. Although the titer is still far below that achieved by natural hosts such as *E. coli* K5 and *E. coli* Nissle 1917 (Suppl. Table 5), it is competitive with, or even superior to, that of other engineered strains including *E. coli* BL21, *Bacillus megaterium* DSM319, and *Bacillus subtilis* 168. Notably, compared with the documented peak titer of 8.02 g/L from engineered *C. glutamicum* (Xue et al. 2026), our overall output still has room for improvement. Nevertheless, our strain achieved a biomass-based heparosan yield of 0.14 g/g DCW, which stands at a relatively advanced level among homologous strains and exhibits excellent biosynthetic capacity. The M_w_ of the heparosan polysaccharide we obtained was approximately 208.42 kDa with a PDI of 1.41. Compared with previous studies (Suppl. Table 5), the heparosan synthesized in the present work features both an elevated molecular weight and a narrow, uniform molecular weight distribution, reflecting sufficient supply of precursor substances and high metabolic flux toward the product synthetic pathway. Furthermore, in contrast to animal-sourced heparin, which exhibits a molecular weight distribution spanning 3–30 kDa (Bertini et al. 2017; van der Meer et al. 2017), the heparosan obtained in this study fully satisfies the prerequisites for subsequent enzymatic modification and controlled depolymerization to prepare pharmaceutical heparin products. However, although the final production capacity was significantly enhanced, cell density remained at a relatively low level compared with data from reported studies (Xue et al. 2026). Accordingly, there remains considerable potential to further enhance the production capacity of Cg24-11 via subsequent fermentation optimization.

## Conclusion

In this study, two complementary strategies were adopted to achieve improved heparosan biosynthesis in the engineered strain Cg24-11. Systematic enhancement of core metabolic fluxes at the genome level drove intracellular carbon source toward activated sugar precursors, establishing sufficient precursor pools in our engineered strain. In parallel, tuning the expression levels of key enzymes via an RBS library facilitated the formation of catalytic complexes, which accelerated the downstream conversion of activated sugar precursors into heparosan. In scale-up experiments using a 5 L bioreactor, Cg24-11 achieved a heparosan titer of 5.36 g/L with M_w_ of 208.42 kDa and PDI of 1.41. The results demonstrate that this complementary strategy yields substantial synergistic effects, jointly promoting heparosan biosynthesis.

## Supplementary Information

Below is the link to the electronic supplementary material.

Supplementary Material 1

## Data Availability

All data generated or analysed during this study are included in this published article (and its supplementary information file).

## Abbreviations

*C. glutamicum*: *Corynebacterium glutamicum*
UDP-GlcA: Uridine diphosphate glucuronic acid
UDP-GlcNAc: Uridine diphosphate N-acetylglucosamine
RBS: Ribosome binding site
SD: Shine-Dalgarno sequence
*E. coli*: *Escherichia coli*
IPTG: Isopropyl β-D-1-thiogalactopyranoside
OD_600_: Optical density at 600 nm
DCW: Dry cell weight
P_*tac*_: Tac promoter
PCR: Polymerase chain reaction
M_w_: Weight-average molecular weight
M_n_: Number-average molecular weight
PDI: Polydispersity index
DO: Dissolved oxygen
UMP: Uridine monophosphate
UDP: Uridine diphosphate
UTP: Uridine triphosphate
