# Supplementary material for: Enhanced heparosan biosynthesis in Corynebacterium glutamicum by systematic metabolic engineering and translation-level fine-tuning
Source: Bioresour Bioprocess. 2026 Aug 17;13(1):114. doi: 10.1186/s40643-026-01113-5 (PMC13478042; doi:10.1186/s40643-026-01113-5)
Supplement: Supplementary file 1 [file 40643_2026_1113_MOESM1_ESM.docx]

**Enhanced heparosan biosynthesis in *Corynebacterium glutamicum* by systematic metabolic engineering and translation-level fine-tuning**

**Supplementary Figures and Tables**

**Figures**

**Suppl. Fig. 1 Schematic diagram of knocking in P*_tac_* upstream of target gene.** The purple part represents the P*_tac_* promoter. The red part represents the target editing region, using for P*_tac_* knocking-in. The green part represents the RBS of target gene. The blue and orange part represent upstream and downstream homology sequences of target editing region, respectively.

**Suppl. Fig. 2 The fluorescence intensity of 384 individual colonies picked from the RBS library.**

**Suppl. Fig. 3 Heparosan molecular weight characterization.** (A) Calibration curve for pullulan standards of different molecular weights. The logarithm of the relative molecular weight of standard samples was plotted against the elution volume (V) of the corresponding chromatographic peaks. Linear regression was performed to establish the molecular weight calibration curve with the following equation: Log MW=25.2−3.45V+0.212V^2^−0.00475V^3^. (B) GPC molecular weight determination of heparosan sample. (C) Heparosan molecular weight characterization.

**Tables**

**Suppl. Table 1 Strains used in this study**

| **Strains** | **Description** | **Sources** |
| --- | --- | --- |
| Cg0 | C*orynebacterium glutamicum* ATCC 13032 with *ldh* and *glgc* knockout | Lab stock |
| Cg1 | Cg0 harboring the plasmid pXMJ19*-kfiA-kfiC* | This study |
| Cg2 | Cg0 harboring the plasmid pXMJ19*-kfiC-kfiA* | This study |
| Cg3 | Cg0 harboring the plasmid pXMJ19*-kfiB-kfiC-kfiA* | This study |
| Cg4 | Cg0 harboring the plasmid pXMJ19*-kfiC-kfiB-kfiA* | This study |
| Cg5 | Cg0 harboring the plasmid pXMJ19*-kfiC-kfiA-kfiB* | This study |
| Cg6 | Cg0 harboring the plasmid pXMJ19*-kfiB-kfiC-kfiA-glmM* | This study |
| Cg7 | Cg0 harboring the plasmid pXMJ19*-kfiB-kfiC-kfiA-ugD* | This study |
| Cg8 | Cg0 harboring the plasmid pXMJ19*-kfiB-kfiC-kfiA-glmU* | This study |
| Cg9 | Cg0 harboring the plasmid pXMJ19*-kfiB-kfiC-kfiA-glmS* | This study |
| Cg10 | Cg0 harboring the plasmid *pXMJ19-kfiB-kfiC-kfiA-galU* | This study |
| Cg11 | Cg0 strain with P*_tac_* inserted upstream of gene *glmM* | This study |
| Cg12 | Cg0 strain with P*_ta_*_c_ inserted upstream of gene *ugD* | This study |
| Cg13 | Cg0 strain with P*_tac_* inserted upstream of gene *glmU* | This study |
| Cg14 | Cg0 strain with P*_tac_* inserted upstream of gene *glmS* | This study |
| Cg15 | Cg0 strain with P*_tac_* inserted upstream of gene *galU* | This study |
| Cg16 | Cg12 strain with P*_tac_* inserted upstream of gene *glmS* | This study |
| Cg17 | Cg12 strain with P*_tac_* inserted upstream of gene *galU* | This study |
| Cg18 | Cg14 strain with P*_tac_* inserted upstream of gene *galU* | This study |
| Cg19 | Cg16 strain with *galE* knockout | This study |
| Cg20 | Cg16 strain with *pyrG* knockout | This study |
| Cg21 | Cg16 strain with *pta* knockout | This study |
| Cg22 | Cg16 strain with *ackA* knockout | This study |
| Cg23 | Cg16 strain with strong RBS inserted upstream of gene *pyrH* | This study |
| Cg24 | Cg16 strain with strong RBS inserted upstream of gene *ndk* | This study |
| Cg24-1 | Cg24 harboring the plasmid p19BCA-rbs1 | This study |
| Cg24-2 | Cg24 harboring the plasmid p19BCA-rbs2 | This study |
| Cg24-3 | Cg24 harboring the plasmid p19BCA-rbs3 | This study |
| Cg24-4 | Cg24 harboring the plasmid p19BCA-rbs4 | This study |
| Cg24-5 | Cg24 harboring the plasmid p19BCA-rbs5 | This study |
| Cg24-6 | Cg24 harboring the plasmid p19BCA-rbs6 | This study |
| Cg24-7 | Cg24 harboring the plasmid p19BCA-rbs7 | This study |
| Cg24-8 | Cg24 harboring the plasmid p19BCA-rbs8 | This study |
| Cg24-9 | Cg24 harboring the plasmid p19BCA-rbs9 | This study |
| Cg24-10 | Cg24 harboring the plasmid p19BCA-rbs10 | This study |
| Cg24-11 | Cg24 harboring the plasmid p19BCA-rbs11 | This study |
| Cg24-12 | Cg24 harboring the plasmid p19BCA-rbs12 | This study |
| Cg24-15 | Cg24 harboring the plasmid p19BCA-rbs15 | This study |

**Suppl. Table 2 Plasmids used in this study**

| **Plasmids** | **Description** | **Sources** |
| --- | --- | --- |
| pXMJ19 | Expression vector of *C. glutamicum*, IPTG-inducible promoter *P_tac_*, Cm^R^ | Lab stock |
| pXMJ19-*kfiA-kfiC* | pXMJ19 derivate, harboring glycosyltransferase gene *kfiA* and *kfiC* from *E. coli* K5 | This study |
| pXMJ19-*kfiC-kfiA* | pXMJ19 derivate, harboring glycosyltransferase gene *kfiA* and *kfiC* from *E. coli* K5 | This study |
| pXMJ19-*kfiB-kfiC-kfiA* | pXMJ19-*kfiC-kfiA* derivate, harboring KfiB coding gene *kfiB,* and the expression cassette was *kfiB-kfiC-kfiA*. | This study |
| pXMJ19-*kfiC-kfiB-kfiA* | pXMJ19-*kfiC-kfiA* derivate, harboring KfiB coding gene *kfiB,* and the expression cassette was *kfiC-kfiB-kfiA*. | This study |
| pXMJ19-*kfiC-kfiA-kfiB* | pXMJ19-*kfiC-kfiA* derivate, harboring KfiB coding gene *kfiB,* and the expression cassette was *kfiC-kfiA-kfiB*. | This study |
| pXMJ19-*kfiB-kfiC-kfiA-glmM* | pXMJ19-*kfiB-kfiC-kfiA* derivate, harboring phosphoglucosamine mutase gene *glmM* | This study |
| pXMJ19-*kfiB-kfiC-kfiA-ugD* | pXMJ19-*kfiB-kfiC-kfiA* derivate, harboring UDP-glucose dehydrogenase gene *ugD* | This study |
| pXMJ19-*kfiB-kfiC-kfiA-glmU* | pXMJ19-*kfiB-kfiC-kfiA* derivate, harboring bifunctional UDP-N-acetylglucosamine pyrophosphorylase / glucosamine-1-phosphate N-acetyltransferase gene *glmU* | This study |
| pXMJ19-*kfiB-kfiC-kfiA-glmS* | pXMJ19-*kfiB-kfiC-kfiA* derivate, harboring L-glutamine-D-fructose-6-phosphate aminotransferase gene *glmS* | This study |
| pXMJ19-*kfiB-kfiC-kfiA-galU* | pXMJ19-*kfiB-kfiC-kfiA* derivate, harboring glucose-1-phosphate uridylyltransferase gene *galU* | This study |
| pK18mobsacB | Suicide vector of *C. glutamicum*, *sacB*-mediated counter-selection, Km^R^ | Lab stock |
| P*_tac_*-M | pK18mobsacB derivate, harboring the upstream and downstream homologous arms for targeted replacement of gene *glmM* and the P*_tac_* promoter sequence, using for P*_tac_* knock-in upstream of gene *glmM*. | This study |
| P*_tac_*-D | pK18mobsacB derivate, harboring the upstream and downstream homologous arms for targeted replacement of gene *ugD* and the P*_tac_* promoter sequence, using for P*_tac_* knock-in upstream of gene *ugD*. | This study |
| P*_tac_*-U | pK18mobsacB derivate, harboring the upstream and downstream homologous arms for targeted replacement of gene *glmU* and the P*_ta_*_c_ promoter sequence, using for P*_tac_* knock-in upstream of gene *glmU*. | This study |
| P*_tac_*-S | pK18mobsacB derivate, harboring the upstream and downstream homologous arms for targeted replacement of gene *glmS* and the P*_tac_* promoter sequence, using for P*_tac_* knock-in upstream of gene *glmS*. | This study |
| P*_tac_*-gU | pK18mobsacB derivate, harboring the upstream and downstream homologous arms for targeted replacement of gene *galU* and the P*_tac_* promoter sequence, using for P*_tac_* knock-in upstream of gene *galU*. | This study |
| pDE-galE | pK18mobsacB derivate, harboring the upstream and downstream homologous arms of *galE* targeted knockout region, using for *galE* knockout. | This study |
| pDE-pryG | pK18mobsacB derivate, harboring the upstream and downstream homologous arms of *pyrG* targeted knockout region, using for *pyrG* knockout. | This study |
| pDE-pta | pK18mobsacB derivate, harboring the upstream and downstream homologous arms of *pta* targeted knockout region, using for *pta* knockout. | This study |
| pDE-ackA | pK18mobsacB derivate, harboring the upstream and downstream homologous arms of *ackA* targeted knockout region, using for *ackA* knockout. | This study |
| pEN-pyrH | pK18mobsacB derivate, harboring the upstream and downstream homologous arms of the RBS of gene *pyrH* and a strong RBS, using for upregulation of *pyrH* | This study |
| pEN-ndk | pK18mobsacB derivate, harboring the upstream and downstream homologous arms of the RBS of gene *ndk* and a strong RBS, using for upregulation of *ndk* | This study |
| pXMJ19-M | pXMJ19 derivate, harboring gene *mCherry* | This study |
| pXMJ19-AM | pXMJ19-M derivate, harboring the RBS of *kfiA* in pXMJ19*-kfiB-kfiC-kfiA* | This study |
| pXMJ19-BM | pXMJ19-M derivate, harboring the RBS of *kfiB* in pXMJ19*-kfiB-kfiC-kfiA* | This study |
| pXMJ19-CM | pXMJ19-M derivate, harboring the RBS of *kfiC* in pXMJ19*-kfiB-kfiC-kfiA* | This study |
| p19BCA-rbsX  (X=1, 2, ...15) | pXMJ19-*kfiB-kfiC-kfiA* derivates, harboring different gene expression cassettes | This study |

**Suppl. Table 3 Primers used in this study**

| **Primers** | **Sequences (5’→3’)** |
| --- | --- |
| AC19-f | ATACGTTGATAGTTTTCATTAACATGCAATATTAATTAAAATATTAC |
| AC19-r | GCTGAATTCTCACTGCTCGATGATACCAGAC |
| V19-AC-F | ATGAAAACTATCAACGTATAAGCTTAATTAATTCTGTTTCCTGTGTG |
| V19-AC-R | CGAGCAGTGAGAATTCAGCTTGGCTGTTTTGG |
| pXMJ19-f | GGAAGCTGTGGTATGGCTGTG |
| pXMJ19-r | CACTTCTGAGTTCGGCATGGG |
| CA19-f | ATTTAGTAAATGCAGTTTTATTCTCATTTTATTTATCATTAAGTG |
| CA19-r | GCTGAATTCTCAGCCTTCCACGTTGTAAACC |
| V19-CA-F | AATTAAGCTTGAATTCAGCTTGGCTGTTTTGGC |
| V19-CA-R | CAAGCTGAATTCAAGCTTAATTAATTCTGTTTCCTGTGTG |
| kfiB-f | CGACAGTGTTTAAAATAAAGTAATCTTGTAGATAATAAAGAGG |
| BC-r | TAAAACTGCATTTACTAAATCTAGCAGCCCTTAATCTTAGCACG |
| BA-r | AATGAAAACTATCAACGTATCTAGCAGCCCTTAATCTTAGCACG |
| BV-r | AGCTGAATTCCTAGCAGCCCTTAATCTTAGCACG |
| VBCA-F | ATTTAGTAAATGCAGTTTTATTCTCATTTTATTTATCATTAAGTGAATGT |
| VBCA-R | CTTTATTTTAAACACTGTCGAAGCTTAATTAATTCTGTTTCCTGTGTG |
| VCBA-F | ATACGTTGATAGTTTTCATTAACATGCAATATTAATTAAAATATTAC |
| VCBA-R | CTTTATTTTAAACACTGTCGTCACTGCTCGATGATACCAGACAC |
| VCAB-F | GGGCTGCTAGGAATTCAGCTTGGCTGTTTTGGC |
| VCAB-R | CTTTATTTTAAACACTGTCGTCAGCCTTCCACGTTGTAAACC |
| VBCA-F | GAATTCAGCTTGGCTGTTTTGGC |
| VBCA-R | TCAGCCTTCCACGTTGTAAACC |
| BCAgU-f | GTTTACAACGTGGAAGGCTGACGATTATCAAAGAGAATTATTTCTAAAATTCGGTATCG |
| BCAgU-r | CAAAACAGCCAAGCTGAATTCTTAAGATTCAAACTCAGCGAGTATTTCCTTCAC |
| BCAD-f | GTTTACAACGTGGAAGGCTGAGACAATCTGAAAACTTGCTGTATCAATAAAACAC |
| BCAD-r | CAAAACAGCCAAGCTGAATTCTTAGTCACGCTGGAAAATATCACGTG |
| BCAS-f | GTTTACAACGTGGAAGGCTGATATTCTCGTCCATTAAGTGATCGAGAAAAAG |
| BCAS-r | CAAAACAGCCAAGCTGAATTCTTATTCGACGGTGACAGACTTTGC |
| BCAM-f | GTTTACAACGTGGAAGGCTGAATAGAGTTTTGAACAATGCGATCTTGG |
| BCAM-r | CAAAACAGCCAAGCTGAATTCTTAGACTTCTGCAACCACTGCAG |
| BCAU-f | GTTTACAACGTGGAAGGCTGATTGCTTGGGTATAAGTGCCCC |
| BCAU-r | CAAAACAGCCAAGCTGAATTCTTAGCCTTCCTGGTTGTGGACG |
| V-SacB-F | TGCCGCAAGCACTCAGG |
| V-SacB-R | GTCATAGCTGTTTCCTGTGTGAAAT |
| SacB-ML-f | ACACAGGAAACAGCTATGACCTGACCTACTTCGCGGCAAG |
| SacB-ML-r | CATTATACGAGCCGATGATTAATTGTCAACCCCAAGAATGCGGAAAACCC |
| SacB-MR-f | AATCATCGGCTCGTATAATGAATGCGATCTTGGCCAGGC |
| SacB-MR-r | CGCCCTGAGTGCTTGCGGCACATTGAGGTCTTCCAGCACGT |
| SacB-DL-f | ACACAGGAAACAGCTATGACGCCATCGCCAGCATGAAATATC |
| SacB-DL-r | CATTATACGAGCCGATGATTAATTGTCAAACTCTCCGACCTCGTTCTAGTT |
| SacB-DR-f | AATCATCGGCTCGTATAATGTGCTGTATCAATAAAACACGAAAAGGAATACT |
| SacB-DR-r | CGCCCTGAGTGCTTGCGGCATCCAGACTTCATTACAAGGCGG |
| SacB-UL-f | ACACAGGAAACAGCTATGACCATCACGGTGATCGTGCTGC |
| SacB-UL-r | AGCCGATGATTAATTGTCAATATTTGGGTGGAAAAGTTGTAAAACTATCCG |
| SacB-UR-f | TTGACAATTAATCATCGGCTCGTATAATGTGCCCCGCAAGAACTTTTAAGG |
| SacB-UR-r | CGCCCTGAGTGCTTGCGGCATCGCCGATGGTCATGTTGG |
| SacB-SL-f | ACACAGGAAACAGCTATGACCGTTAGCGGTTCTCTACGTG |
| SacB-SL-r | CATTATACGAGCCGATGATTAATTGTCAAAGCAGTGACACCAGTCAGC |
| SacB-SR-f | AATCATCGGCTCGTATAATGAGTGATCGAGAAAAAGTTGTTGTAAAGTC |
| SacB-SR-r | CGCCCTGAGTGCTTGCGGCAGAGCAACCTGGCCTGCATAAG |
| SacB-gUL-f | ACACAGGAAACAGCTATGACCATCACGGTGATCGTGCTGC |
| SacB-gUL-r | AGCCGATGATTAATTGTCAATATTTGGGTGGAAAAGTTGTAAAACTATCCG |
| SacB-gUR-f | TTGACAATTAATCATCGGCTCGTATAATGTGCCCCGCAAGAACTTTTAAGG |
| SacB-gUR-r | CGCCCTGAGTGCTTGCGGCATCGCCGATGGTCATGTTGG |
| SacB-f | GAGTGAGCTGATACCGCTCG |
| SacB-r | CCAGATAGCCCAGTAGCTGAC |
| De-galE-Lf | CACAGGAAACAGCTATGACGGCGTGAGATGACAGATCAGG |
| De-galE-Lr | GCATTGGTGCGTGTGTTAGCCCTCAACCTTTTCTAC |
| De-galE-Rf | GCTAACACACGCACCAATGCTTATGGCGCAAC |
| De-galE-Rr | CGCCCTGAGTGCTTGCGGCACATTGATTCATCATCTGGCGCC |
| De-pyrG-Lf | CACAGGAAACAGCTATGACCGCGGACCAGCTGAAATCAATC |
| De-pyrG-Lr | CTGGTTCGCCGATGTCTGAGACGACCTTCCTGG |
| De-pyrG-Rf | CTCAGACATCGGCGAACCAGATGCTCATGG |
| De-pyrG-Rr | CGCCCTGAGTGCTTGCGGCACAGTTCCGCTACTAAGGAGCC |
| De-pta-Lf | CACAGGAAACAGCTATGACCAACGTCCAACGCCACGTTAC |
| De-pta-Lr | CTGGTTCGAGCATCGCCTTTCTAATTTCAGCCTG |
| De-pta-Rf | GAAAGGCGATGCTCGAACCAGTCATGAGCGC |
| De-pta-Rr | CGCCCTGAGTGCTTGCGGCACTGTGTTGACGATGTCAGGGAC |
| De-ackA-Lf | CACAGGAAACAGCTATGACCAGAGCACTCCCACATTGTGC |
| De-ackA-Lr | CAATGCCGTCATTAGCTGCGTCCTCCTGCC |
| De-ackA-Rf | CGCAGCTAATGACGGCATTGATGTTGCTCG |
| De-ackA-Rr | CGCCCTGAGTGCTTGCGGCACAACAAGCGATTCCTGTGAAGC |
| En-ndk-Lf | CACAGGAAACAGCTATGACGAGCGTGACTGATGGAAACGTC |
| En-ndk-Lr | CATTATGGGCACTCCTTTCTAATGGTGGTCTGCTTCGCG |
| En-ndk-Rf | GAAAGGAGTGCCCATAATGACTGAACGTACTCTCATCCTTATCAAG |
| En-ndk-Rr | CGCCCTGAGTGCTTGCGGCACAACTGCACGACCAGAAACGAG |
| En-pyrH-Lf | CACAGGAAACAGCTATGACCTGGCAACACCATGGTCGAG |
| En-pyrH-Lr | CACTATGGGCACTCCTTTTCAGTAGGGGTTGTGCCAACC |
| En-pyrH-Rf | GAAAAGGAGTGCCCATAGTGACCACCTCGAGTGAACAAC |
| En-pyrH-Rr | CGCCCTGAGTGCTTGCGGCAGGCACGCCGTAGTATTCAGC |
| M-F1 | GCTGGCAGTTCTAGACCGAC |
| M-F2 | GACTTGGGTTTACACAGCTCAACC |
| M-R | GCCAGTGGTGCCATGATCTG |
| D-F1 | GCTGGCAGAATTCTCCTAATCCG |
| D-F2 | CCTTCGATAAAACAAAGACAATTTTCCCC |
| D-R | CTCATCGGTTGGACGATTTGC |
| U-F1 | GCTTCTGGTTCCAATCCGCG |
| U-F2 | GCTTGCTTGGGTATAAGTGCC |
| U-R | CTACGAAGCCACCGAGCTTG |
| S-F1 | CACGGCGATTTGATCATTCCAG |
| S-F2 | CTCAGTGTGCTGTGTGGATTG |
| S-R | GGACTGGGACAATGCCACAAC |
| gU-F1 | GCTAAGACTCTTGAGGTCACCG |
| gU-F2 | GCTGGTTAGTGCTCATACTCACAC |
| gU-R | CCTCCCACAATGTGGAAGGC |
| De-galE-F1 | CATGAGTAAGCTCCGCGATGG |
| De-galE-F2 | GATTCGAGGGTGTCGTCCAC |
| De-galE-R | GATGTACTTACCCGTGCGCTC |
| De-pyrG-F1 | GTCATGGCTGTTGCAGCAAG |
| De-pyrG-F2 | CCACTGTGATCGCCAAGGAG |
| De-pyrG-R | CGCGGTAGGCATTATTGACC |
| De-pta-F1 | GCTCCAACGGAGTGAACTTCG |
| De-pta-F2 | GCCAGCTCTCATCATCGGTG |
| De-pta-R | GTGCCAATGCCATTAGCTGC |
| De-ackA-F1 | CCTCGAACCAGTCATGAGCG |
| De-ackA-F2 | CATCGCAGATCACTCCGAAGG |
| De-ackA-R | GATGTGATTTCTGCGGGGTTACTG |
| V19M-F | CAAGTAAGAATTCAGCTTGGCTGTTTTGG |
| V19M-R | CGACACCATAAGCTTAATTAATTCTGTTTCCTGTGTG |
| P19M-f | GAATTAATTAAGCTTATGGTGTCGAAGGGCGAAG |
| P19M-r | CCAAGCTGAATTCTTACTTGTACAGTTCATCCATGCCTCCAG |
| RBS-wk1-f | GAAACAGAAAGGAGNNNNNNATGGTGTCGAAGGGCGAAG |
| RBS-wk1-r | CACCATNNNNNNCTCCTTTCTGTTTCCTGTGTGAAATTGTTATCCG |
| RBS-wk2-f | GAAACAGAAAGGAGNNNNNNNATGGTGTCGAAGGGCGAAG |
| RBS-wk2-r | CACCATNNNNNNNCTCCTTTCTGTTTCCTGTGTGAAATTGTTATCCG |
| RBS-wk3-f | GAAACAGAAAGGAGNNNNNNNNATGGTGTCGAAGGGCGAAG |
| RBS-wk3-r | CACCATNNNNNNNNCTCCTTTCTGTTTCCTGTGTGAAATTGTTATCCG |
| 19-AM-F | GATAGTTTTCATTAACATGCAATATTAATTAAAATATTACCCCATGGTGTCGAAGGGCGAAG |
| 19-AM-R | GCATGTTAATGAAAACTATCAACGTATAAGCTTAATTAATTCTGTTTCCTGTGTGAAATTG |
| 19-BM-F | GTTTAAAATAAAGTAATCTTGTAGATAATAAAGAGGAAATATGATGGTGTCGAAGGGCGAAG |
| 19-BM-R | CAAGATTACTTTATTTTAAACACTGTCGAAGCTTAATTAATTCTGTTTCCTGTGTGAAATTG |
| 19-CM-F | GTAAATGCAGTTTTATTCTCATTTTATTTATCATTAAGTGAATGTATGGTGTCGAAGGGCGAAG |
| 19-CM-R | GAGAATAAAACTGCATTTACTAAATAAGCTTAATTAATTCTGTTTCCTGTGTGAAATTG |
| V19rbs-F | GGAAGGCTGAGAATTCAGCTTGGCTGTTTTGGC |
| V19rbs-R | CTCCTTTACTGTCGAAGCTTAATTAATTCTGTTTCCTGTGTG |
| PkfiB-1f | TAAGCTTCGACAGTAAAGGAGTGCCCATAATGAACAAGCTGGTGCTCGTG |
| PkfiB-rrr | CTCCTTTACTAAATCTAGCAGCCCTTAATCTTAGCACG |
| PkfiC-1f | GCTAGATTTAGTAAAGGAGCAGCACAAATGAACGCAGAGTACATCAACCTG |
| PkfiC-rrr | CTCCTTTAACGTATTCACTGCTCGATGATACCAGACAC |
| PkfiA-1f | CAGTGAATACGTTAAAGGAGAAAGCCACATGATCGTCGCAAACATGTCCAG |
| PkfiA-rrr | GCTGAATTCTCAGCCTTCCACGTTGTAAACC |
| PkfiB-2f | TAAGCTTCGACAGTAAAGGAGACCAATATGAACAAGCTGGTGCTCGTG |
| PkfiC-2f | GCTAGATTTAGTAAAGGAGTATTAAATATGAACGCAGAGTACATCAACCTG |
| PkfiA-2f | CAGTGAATACGTTAAAGGAGTGCAATCTATGATCGTCGCAAACATGTCCAG |
| PkfiB-3f | TAAGCTTCGACAGTAAAGGAGTAAACAATGAACAAGCTGGTGCTCGTG |
| PkfiC-3f | GCTAGATTTAGTAAAGGAGTAACCCTGATGAACGCAGAGTACATCAACCTG |
| PkfiA-3f | CAGTGAATACGTTAAAGGAGCAAACAAATGATCGTCGCAAACATGTCCAG |
| PkfiB-4f | TAAGCTTCGACAGTAAAGGAGTGTGGCCATGAACAAGCTGGTGCTCGTG |
| PkfiC-4f | GCTAGATTTAGTAAAGGAGCTCTATTTATGAACGCAGAGTACATCAACCTG |
| PkfiA-4f | CAGTGAATACGTTAAAGGAGAGTACCATGATCGTCGCAAACATGTCCAG |
| PkfiB-5f | TAAGCTTCGACAGTAAAGGAGCTCTATTTATGAACAAGCTGGTGCTCGTG |
| PkfiC-5f | GCTAGATTTAGTAAAGGAGCTATTTATGAACGCAGAGTACATCAACCTG |
| PkfiA-5f | CAGTGAATACGTTAAAGGAGTGTGGCCATGATCGTCGCAAACATGTCCAG |
| PkfiB-6f | TAAGCTTCGACAGTAAAGGAGCTACAGAGATGAACAAGCTGGTGCTCGTG |
| PkfiC-6f | GCTAGATTTAGTAAAGGAGTAATACATGAACGCAGAGTACATCAACCTG |
| PkfiA-6f | CAGTGAATACGTTAAAGGAGAGAGGAAATGATCGTCGCAAACATGTCCAG |
| PkfiB-7f | TAAGCTTCGACAGTAAAGGAGAGGAGCAATGAACAAGCTGGTGCTCGTG |
| PkfiC-7f | GCTAGATTTAGTAAAGGAGCTACAGAGATGAACGCAGAGTACATCAACCTG |
| PkfiA-7f | Same asPkfiA-6f |
| PkfiB-8f | TAAGCTTCGACAGTAAAGGAGGACAGGATGAACAAGCTGGTGCTCGTG |
| PkfiC-8f | GCTAGATTTAGTAAAGGAGACTTGCATGAACGCAGAGTACATCAACCTG |
| PkfiA-8f | CAGTGAATACGTTAAAGGAGGCGACGCATGATCGTCGCAAACATGTCCAG |
| PkfiB-9f | TAAGCTTCGACAGTAAAGGAGGTATAGAAATGAACAAGCTGGTGCTCGTG |
| PkfiC-9f | GCTAGATTTAGTAAAGGAGAATCCTACATGAACGCAGAGTACATCAACCTG |
| PkfiA-9f | CAGTGAATACGTTAAAGGAGGGAGACATGATCGTCGCAAACATGTCCAG |
| PkfiB-10f | TAAGCTTCGACAGTAAAGGAGCAGCACAAATGAACAAGCTGGTGCTCGTG |
| PkfiC-10f | GCTAGATTTAGTAAAGGAGAGGCGTAATGAACGCAGAGTACATCAACCTG |
| PkfiA-10f | CAGTGAATACGTTAAAGGAGTAATACATGATCGTCGCAAACATGTCCAG |
| PkfiB-11f | TAAGCTTCGACAGTAAAGGAGAGGCGTAATGAACAAGCTGGTGCTCGTG |
| PkfiC-11f | Same as PkfiC-1f |
| PkfiA-11f | Same as PkfiA-10f |
| PkfiB-12f | Same as PkfiB-11f |
| PkfiC-12f | Same as PkfiC-6f |
| PkfiA-12f | CAGTGAATACGTTAAAGGAGCAGCACAAATGATCGTCGCAAACATGTCCAG |
| PkfiB-13f | Same as PkfiB-3f |
| PkfiC-13f | Same as PkfiC-10f |
| PkfiA-13f | Same as PkfiA-10f |
| PkfiB-14f | Same as PkfiB-11f |
| PkfiC-14f | GCTAGATTTAGTAAAGGAGTAAACAATGAACGCAGAGTACATCAACCTG |
| PkfiA-14f | Same as PkfiA-10f |
| PkfiB-15f | Same as PkfiB-11f |
| PkfiC-15f | Same as PkfiC-6f |
| PkfiA-15f | CAGTGAATACGTTAAAGGAGTAAACAATGATCGTCGCAAACATGTCCAG |

**Suppl. Table 4 RBS sequences with different translation efficiencies**

| **RBS** | **Sequences (5’→3’)** |
| --- | --- |
| R1 | (AGGAG) **CAGCACAA** |
| R2 | (AGGAG) **TGCCCATA** |
| R3 | (AGGAG) **AAAGCCAC** |
| R4 | (AGGAG) **CAGTAAC** |
| R5 | (AGGAG) **TAACCCTG** |
| R6 | (AGGAG) **TATTAAAT** |
| R7 | (AGGAG) **TAAACA** |
| R8 | (AGGAG) **ACCAAT** |
| R9 | (AGGAG) **CAAACAA** |
| R10 | (AGGAG) **TGCAATCT** |
| R11 | (AGGAG) **CTATTT** |
| R12 | (AGGAG) **CAGGTAT** |
| R13 | (AGGAG) **CTCTATTT** |
| R14 | (AGGAG) **TGGACCT** |
| R15 | (AGGAG) **TGTGGCC** |
| R16 | (AGGAG) **CGGACCG** |
| R17 | (AGGAG) **AGTACC** |
| R18 | (AGGAG) **ATATCT** |
| R19 | (AGGAG) **AGTCCT** |
| R20 | (AGGAG) **TACCAGC** |
| R21 | (AGGAG) **AGGCGTA** |
| R22 | (AGGAG) **TAATAC** |
| R23 | (AGGAG) **CTACAGAG** |
| R24 | (AGGAG) **AGGAGCA** |
| R25 | (AGGAG) **AGAGGAA** |
| R26 | (AGGAG) **ACTTGC** |
| R27 | (AGGAG) **GACAGG** |
| R28 | (AGGAG) **GCGACGC** |
| R29 | (AGGAG) **AATCCTAC** |
| R30 | (AGGAG) **GTATAGAA** |
| R31 | (AGGAG) **GGAGAC** |
| R32 | (AGGAG) **GTTACAGT** |

Bracketed sequences indicate identical SD sequences, while bolded segments represent the spacer sequences of specific RBS.

**Suppl. Table 5 Summary of literatures on heparosan biosynthesis**

| **Strains** | **Biosynthetic strategy** | **Heparosan titer** | **Molecular weight** | **References** |
| --- | --- | --- | --- | --- |
| *E. coli* K5 | Fermenter parameter optimization | 15 g/L  (7 L fed-batch) | M_w_=84 kDa; PDI=1.45 | (Wang et al. 2010) |
| *E. coli* Nissle 1917 | Screen and assemble key genes (*galU*, *kfiD* and *glmM*) of different origins; insert translational enhancers for *kfiA* and *kfiC* overexpression | 11.5 g/L  (3 L fed-batch） | M_w_=624.52 kDa; PDI=1.86 | (Hu et al. 2022) |
| *E. coli* Nissle 1917 | Increase copy number of chromosomal *kps* gene cluster | 9.1 g/L  (5 L fed-batch) | Not determined | (Yu et al. 2023) |
| *E. coli* Nissle 1917 | Optimize the expression of *kfiA*, *kfiB* and *kfiC*; overexpress precursor synthesis and transport genes | 12.2 g/L  (5 L fed-batch) | M_w_=705.73 kDa; PDI=1.18 | (Hu et al. 2024) |
| *E. coli* BL21 | Co-overexpression of *kfiA*, *kfiB*, *kfiC*, and *kfiD* together with fermenter optimization | 1.88 g/L  (3 L fed-batch) | M_w_=118.30 kDa; PDI=1.04 | (Zhang et al. 2012) |
| *E. coli* BL21 | TF replaces KfiB to stabilize KfiA-KfiC complex | 1.5 g/L  (0.5 L fed-batch) | M_w_=67.92 kDa; PDI=1.78 | (Leroux and Priem 2016) |
| *B. megaterium* DSM319 | Overexpression of *tuaD* and *gtaB* together with fermentation optimization | 1.96 g/L  (3 L fed-batch) | M_w_=41.9 kDa | (Nehru et al. 2021) |
| *B. subtilis* 168 | Overexpression of *tuaD* | 5.82 g/L  (3 L fed-batch) | M_w_=58.25 kDa; PDI=1.91 | (Jin et al. 2016) |
| *C. glutamicum* ATCC 13032 | Fermenter parameter optimization | 8.02 g/L  (5 L fed-batch) | M_w_=5.9 kDa | (Xue et al. 2026) |
|  |  | **Titer / DCW＜0.12 g/g DCW** | |  |
| *C. glutamicum* ATCC 13032 | Genomic-level modulation together with translation-level fine-tuning | 5.36 g/L  (5 L fed-batch) | M_w_=208.42 kDa;  PDI=1.41 | This study |
|  |  | **Titer / DCW=0.14 g/g DCW** | |  |

**References**

Hu S, Zhao L, Hu L et al (2022) Engineering the probiotic bacterium *Escherichia coli* Nissle 1917 as an efficient cell factory for heparosan biosynthesis. Enzyme Microb Technol 158:110038. <https://doi.org/10.1016/j.enzmictec.2022.110038>

Hu S, Zhou SY, Wang Y et al (2024) Coordinated optimization of the polymerization and transportation processes to enhance the yield of exopolysaccharide heparosan. Carbohydr Polym 333:121983. <https://doi.org/10.1016/j.carbpol.2024.121983>

Jin P, Zhang LP, Yuan PH et al (2016) Efficient biosynthesis of polysaccharides chondroitin and heparosan by metabolically engineered *Bacillus subtilis*. Carbohydr Polym 140:424-432. <https://doi.org/10.1016/j.carbpol.2015.12.065>

Leroux M, Priem B (2016) Chaperone-assisted expression of KfiC glucuronyltransferase from *Escherichia coli* K5 leads to heparosan production in *Escherichia coli* BL21 in absence of the stabilisator KfiB. Appl Microbiol Biotechnol 100(24):10355-10361. <https://doi.org/10.1007/s00253-016-7745-8>

Nehru G, Tadi SRR, Sivaprakasam S (2021) Application of dual promoter expression system for the enhanced heparosan production in *Bacillus megaterium*. Appl Biochem Biotechnol 193(8):2389-2402. <https://doi.org/10.1007/s12010-021-03541-9>

Wang ZY, Ly M, Zhang FM et al (2010) *E. coli* K5 fermentation and the preparation of heparosan, a bioengineered heparin precursor. Biotechnol Bioeng 107(6):964-973. <https://doi.org/10.1002/bit.22898>

Xue Q, Wu R, Yu J et al (2026) Systematic optimization of fed-batch fermentation for enhanced heparosan production in engineered *Corynebacterium glutamicum*. Int J Biol Macromol 338(Part 1):149687. <https://doi.org/10.1016/j.ijbiomac.2025.149687>

Yu YY, Gong BX, Wang HL et al (2023) Chromosome evolution of Escherichia coli Nissle 1917 for high-level production of heparosan. Biotechnol Bioeng 120(4):1081-1096. <https://doi.org/10.1002/bit.28315>

Zhang CY, Liu L, Teng LP et al (2012) Metabolic engineering of *Escherichia coli* BL21 for biosynthesis of heparosan, a bioengineered heparin precursor. Metab Eng 14(5):521-527. <https://doi.org/10.1016/j.ymben.2012.06.005>
